# Metabolic Reprogramming Driven by Trophoblasts and Decidual XCR1^+^PMN‐MDSC Crosstalk Controls Adverse Outcomes Associated With Advanced Maternal Age

**DOI:** 10.1002/advs.202513370

**Published:** 2026-01-12

**Authors:** Meiqi Chen, Yuxiong Guo, Qing Zhao, Jingping Liu, Shuyi Kuang, Zhengcong Huang, Chenlin Lv, Shuxiu Xu, Zekai Zhuang, Anyan Yang, Jing Li, Kai Wu, Yumei He

**Affiliations:** ^1^ Pediatric Intensive Care Unit Guangdong Provincial People's Hospital (Guangdong Academy of Medical Sciences) Department of Immunology School of Basic Medical Sciences Department of Clinical Laboratory The Third Affiliated Hospital of Southern Medical University Southern Medical University Guangzhou China; ^2^ Department of Immunology School of Basic Medical Sciences Guangdong Provincial Key Laboratory of Single‐cell and Extracellular Vesicles Southern Medical University Guangzhou China; ^3^ Pediatric Intensive Care Unit Guangdong Provincial Cardiovascular Institute Guangdong Provincial People's Hospital (Guangdong Academy of Medical Sciences) Southern Medical University;Guangdong Provincial People's Hospital Guangdong Academy of Medical Sciences Guangzhou China; ^4^ Department of Clinical Laboratory The Third Affiliated Hospital of Southern Medical University Southern Medical University Guangzhou China; ^5^ Department of Obstetrics and Gynecology Nanfang Hospital Southern Medical University Guangzhou China; ^6^ Department of Pediatric Surgery Zhujiang Hospital Southern Medical University Guangzhou China

**Keywords:** advanced maternal age, adverse pregnancy outcomes, crosstalk, metabolic reprogramming, polymorphonuclear myeloid‐derived suppressor cells

## Abstract

Trophoblast–immune cell communication is crucial during pregnancy, with impairments linked to adverse outcomes. The accumulation of decidual polymorphonuclear myeloid‐derived suppressor cells (dPMN‐MDSCs) in the third trimester is vital for fetal development. This study presents a novel crosstalk mechanism between trophoblasts and dPMN‐MDSCs that improves adverse outcomes associated with advanced maternal age (AMA). A specific dPMN‐MDSC population with high X‐C motif chemokine receptor 1 (XCR1) expression is identified, which interacts with trophoblasts through X‐C motif chemokine ligand 1 (XCL1) during the third trimester. Spontaneous fetal growth restriction observed in AMA and pregnant *Xcr1^−/−^* mice is correlated with the disruption of this interaction. Mechanistically, the deficiency in XCL1–XCR1 expression reduces nuclear FOXO1 levels, thereby impairing the transcription of FOXO1‐driven oxidative phosphorylation genes in decidual XCR1^+^PMN‐MDSCs. Restoring the expression of XCL1–XCR1 or FOXO1 in dPMN‐MDSCs mitigates this effect. Crucially, their adoptive transfer or treatment with XCL1/Oltipraz rescues the delayed fetal growth linked to impaired decidual XCR1^+^PMN‐MDSCs and metabolic imbalance. Our findings highlight the importance of trophoblast–dPMN‐MDSC communication via the XCL1–XCR1 axis, proposing metabolic reprogramming of dPMN‐MDSCs as a potential immunotherapeutic strategy for AMA‐related adverse outcomes.

## Graphical abstract

The interaction between trophoblasts and decidual polymorphonuclear myeloid‐derived suppressor cells (dPMN‐MDSCs) via the XCL1–XCR1 axis is crucial for fetal development during the third trimester. Disruption of this axis impairs FOXO1 activity and causes metabolic imbalance in dPMN‐MDSCs, contributing to adverse outcomes associated with advanced maternal age (AMA).

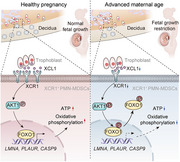

## 1 Introduction

As the leading cause of maternal death, adverse pregnancy outcomes pose serious consequences during the perinatal period, negatively impacting the long‐term health of the mother and fetus [1, 2]. Adverse perinatal outcomes, which affect at least 30% of pregnant women, include preeclampsia (PE), gestational diabetes mellitus (GDM), stillbirth, preterm birth (PTB), and fetal growth restriction (FGR) [3, 4]. The causes of these outcomes encompass non‐pathological factors, such as genetics and environmental influences, as well as pathological factors, such as placental dysfunction and maternal immunological changes [5, 6]. In recent years, instances of advanced maternal age (AMA; i.e., ≥ 35 years of age) have increased worldwide and received considerable attention as an independent risk factor for adverse pregnancy outcomes [7, 8], necessitating research to address this global challenge.

Successful pregnancy involves the function and coordination of decidual immune cells (DICs), decidual stromal cells (DSCs), and trophoblasts [9, 10], supporting maternal–fetal tolerance and the health of the growing fetus. Previous studies have suggested that these cells function independently at the maternal–fetal interface; however, recent evidence shows that DICs can be uniquely activated and regulated by trophoblasts through interaction [11, 12]. Specifically, CXCR4^+^ decidual natural killer (dNK) cells are recruited and reprogrammed by trophoblasts during the first trimester, facilitating a type‐2 T helper cell (Th2) bias and a low‐cytotoxic phenotype [13]. Interleukin (IL)‐35 secreted by trophoblasts inhibits the proliferation of human naïve conventional T cells and induces their conversion into iT_R_35 cells, which are essential for successful pregnancy [14]. Moreover, trophoblast‐derived CXCL16 favors the polarization and anti‐inflammatory behavior of decidual macrophages (dMφ) and restricts NK cell activation and cytotoxicity [15]. Notably, the X‐C motif chemokine ligand 1 (XCL1)/X‐C motif chemokine receptor 1 (XCR1) pathway is critical for enhancing trophoblastic function in the first trimester [16]; however, its effects during the third trimester remain unclear.

DIC abundance varies during pregnancy [17], particularly in the case of decidual polymorphonuclear myeloid‐derived suppressor cells (dPMN‐MDSCs), which continuously accumulate and peak during the third trimester [18]. The connection between dPMN‐MDSCs and adverse pregnancy outcomes underscores the importance of this cell population. For example, TRAIL upregulation and DcR2 downregulation in dPMN‐MDSCs have been shown to promote apoptosis and reduce PMN‐MDSC levels, which are related to unexplained recurrent pregnancy loss [19]. Additionally, hypoxia‐inducible factor 1α (HIF‐1α) deficiency in myeloid cells results in diminished MDSC expansion and immunosuppression, leading to an increased abortion rate in mice [20]. We previously demonstrated that dPMN‐MDSCs produce growth‐promoting factors (GPFs) to support fetal growth but are impaired in FGR [21]. Notably, the number and function of dPMN‐MDSCs appear to be correlated with trophoblast‐induced communication [22, 23]. However, data on the mechanisms underlying this crosstalk and their regulation of adverse outcomes associated with AMA remain limited.

Immune cells are known to respond to specific surrounding metabolic changes, such as hypoxia, nutrient availability, mitochondrial stress, and exogeneous stimuli [24, 25]. These behaviors lead to metabolic reprogramming to support the bioenergetic and biosynthetic requirements of activation, cellular activity, and functional remodeling [26, 27]. In mitochondria, multiple signaling pathways metabolize metabolites via the tricarboxylic acid (TCA) cycle and oxidative phosphorylation (OXPHOS), playing vital roles in metabolic reprogramming [28, 29]. Caspase 9 (CASP9), lamin A/C (LMNA), and plasminogen activator receptor (PLAUR) are key regulators of cellular metabolism that regulate mitochondrial function and affect OXPHOS [30, 31, 32, 33]. In low‐oxygen environments, PMN‐MDSC expansion and function are maintained through metabolic process; activated glycolysis and elevated lactate release in PMN‐MDSCs contribute to their functionality [34]. Intracellular lipid accumulation and increased arachidonic acid metabolism drive the differentiation of circulating neutrophils into dPMN‐MDSCs during the first trimester [35]; however, the precise metabolic changes triggered by the decidual microenvironment and their roles in adverse outcomes caused by AMA remain unknown.

In this study, human samples and mouse models were used to demonstrate a novel crosstalk mechanism between trophoblasts and dPMN‐MDSCs via the XCL1–XCR1 axis, with disruption associated with adverse outcomes in AMA. The strong positive correlation between this axis and fetal development provides a valuable theoretical basis for targeting XCL1‐activated decidual XCR1^+^PMN‐MDSCs to prevent AMA‐related adverse outcomes.

## 2 Results

### 2.1 Impaired Expression of the XCL1–XCR1 Axis is Linked to Reduced dPMN‐MDSC Abundance and Function in AMA

**FIGURE 1:**
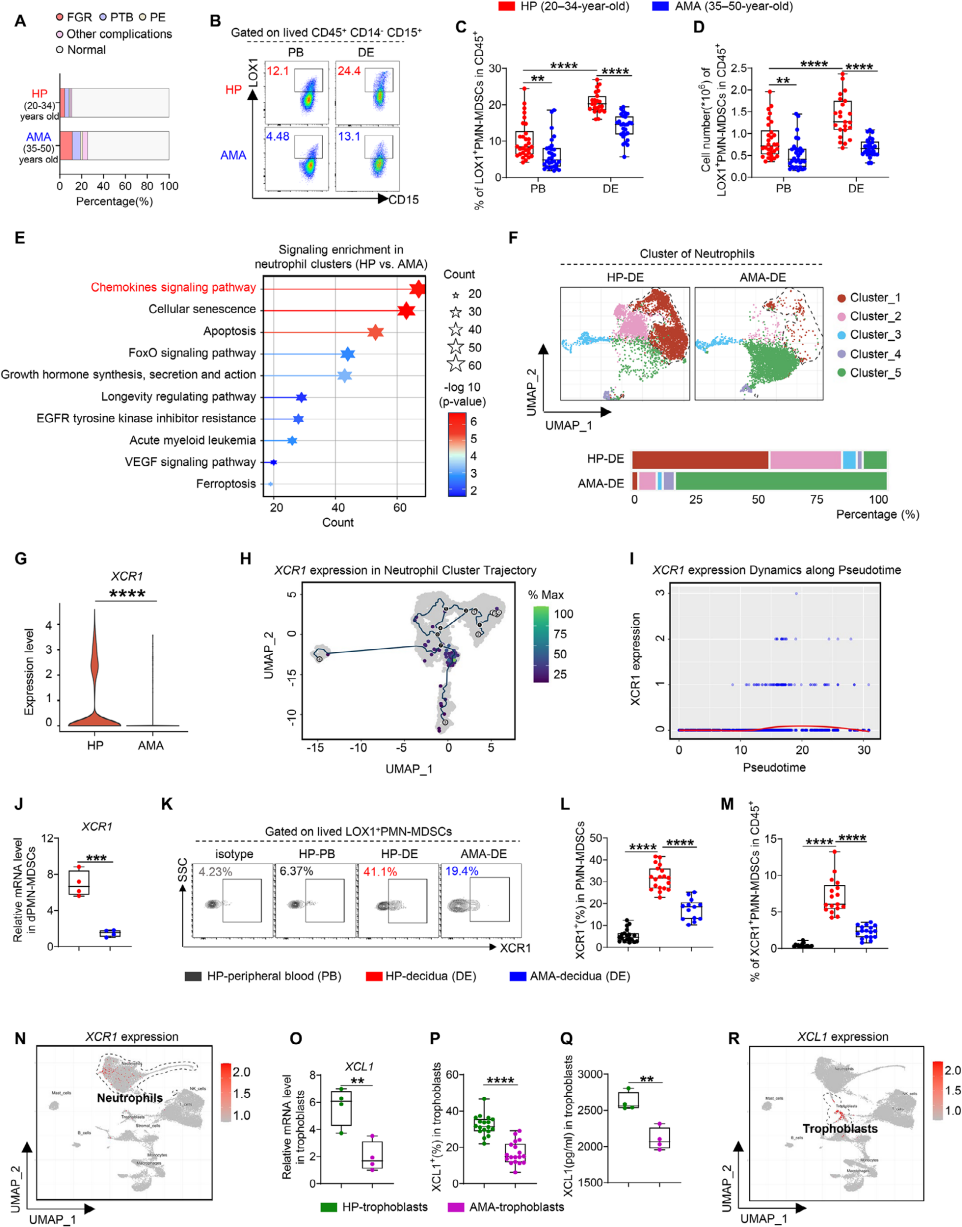
Impaired expression of the XCL1–XCR1 axis is linked to reduced dPMN‐MDSC abundance and function in advanced maternal age (AMA). (A) Incidence of pregnancy complications in healthy pregnancies (HP) and AMA (*n =* 200). (B–D) Representative flow cytometry plots and statistical analysis of LOX1^+^PMN‐MDSCs among CD45^+^ cells (peripheral blood, PB, *n =* 30. decidua, DE, HP, *n =* 23. AMA, *n =* 28). (E) Enriched signaling pathways of differentially expressed genes (DEGs) in neutrophils between the HP and AMA groups. (F) Uniform Manifold Approximation and Projection (UMAP) visualizing the neutrophil subclusters and proportion of each cluster (HP, *n =* 4. AMA, *n =* 3). (G) *XCR1* expression in neutrophil cluster_1. (H) *XCR1* expression pattern overlaid on the pseudotime trajectory of neutrophil clusters. (I) Dynamic changes in *XCR1* expression along the inferred development path of neutrophil clusters. (J) mRNA expression level of *XCR1* in decidual PMN‐MDSCs (dPMN‐MDSCs) (*n =* 4 replicates). (K–M) Representative flow cytometry plots (K), percentages of XCR1^+^ cells in PMN‐MDSCs (L, HP, *n* = 19. AMA, *n* = 13), and statistical analysis of XCR1^+^PMN‐MDSCs (M, *n* = 17). (N) Distribution of *XCR1* expression among human decidua. (O–Q) mRNA expression levels (O, *n =* 4 replicates), percentages (P, *n* = 18), and secretion levels (Q, *n* = 4) of XCL1 in trophoblasts. (R) Distribution of *XCL1* expression among human decidua. Data are presented as mean ± SEM. Each dot represents a single individual. ns, not significant; ^*^ *p* < 0.05, ^**^ *p* < 0.01, ^***^ *p* < 0.001, ^****^ *p* < 0.0001. Statistical significance was determined using a Student's *t‐*test (C, D, J, L, and O–Q), Mann–Whitney test (C, D, and M), or Wilcoxon test (G).

Given the well‐documented association between AMA and adverse outcomes, we sought to investigate whether dPMN‐MDSCs—previously linked to FGR [21]—are functionally impaired in AMA and to identify the underlying molecular pathways.​ To this end, we first collected clinical data from 400 patients stratified into two groups: healthy pregnancies (HP) and AMA (n = 200 per group). Clinical analyses revealed that women with AMA exhibited a higher incidence of adverse pregnancy outcomes than those in the HP group, particularly for FGR, which occurred in 11.5% (AMA) vs. 4.5% (HP) (Figure 1A). Notably, the abundance of LOX1^+^PMN‐MDSCs in peripheral blood (pPMN‐MDSCs) decreased at each stage of AMA, with particular declines during the third trimester (Figure S1A). Furthermore, pPMN‐MDSC activation was diminished in AMA, as evidenced by reduced immunosuppressive activity and the downregulation of related targets and transcription factor STAT6 (Figure S1B–F). Notably, while PMN‐MDSCs were more prevalent in the decidua (dPMN‐MDSCs) during the third trimester, both their level and number declined significantly in AMA (Figure 1B–D). Crucially, multivariate linear regression analyses showed a significant negative correlation between dPMN‐MDSC levels and maternal age (Figure S2A). However, the sex of offspring from AMA mothers did not affect dPMN‐MDSC levels or fetal growth parameters (Figure S2B–G). Furthermore, no significant increase in inflammation was observed in the circulation or placental tissues of AMA compared to the HP group (Figure S2H–J). RNA sequencing (RNA‐seq) revealed distinct gene expression profiles in dPMN‐MDSCs compared to pPMN‐MDSCs, with significant enrichments in the chemokine signaling pathway (Figure S3A,B). qPCR results further confirmed a higher expression of the chemokine receptors *XCR1* and *CMKLR2*, as well as GPFs, such as osteopontin (OPN, encoded by *SPP1*), osteoglycin (OGN), and pleiotrophin (PTN) in dPMN‐MDSCs (Figure S3C,D). These results demonstrate that dPMN‐MDSCs are functionally compromised in cases of AMA, with chemokine signaling emerging as a potential regulatory pathway.

Based on the recognition of chemokine signaling as critical in dPMN‐MDSCs and XCR1 as a prominent receptor, we examined dPMN‐MDSC heterogeneity at single‐cell resolution and validated XCR1 specificity. To achieve this, single‐cell RNA‐sequencing (scRNA‐seq) was performed on LOX1^+^ immune cells sorted from decidual basalis samples of HP and AMA groups. Following quality control, graph‐based clustering, and classic marker gene annotation (Figure S4A), we obtained a map of 28,821 meta‐cells representing nine distinct cell types, with neutrophils as the predominant population (Figure S4B). Kyoto Encyclopedia of Genes and Genomes (KEGG) analysis revealed the enrichment of chemokine signaling pathways within the neutrophil cluster (Figure 1E). This neutrophil population was subsequently divided into five clusters using an unsupervised clustering and molecular signature analysis (Figure S4C). Uniform Manifold Approximation and Projection (UMAP) revealed that, among these five clusters, neutrophil cluster_1 was significantly reduced in the AMA group (Figure 1F). Within the chemokine signaling pathway, *XCR1* expression in neutrophil cluster_1 was prominently downregulated in the AMA group (Figure 1G). In contrast, no significant difference was observed in *CCR1* expression (another receptor in the chemokine signaling pathway) between the HP and AMA groups (Figure S4D,E). Pseudotime trajectory analysis revealed a clear and continuous developmental path for the neutrophil clusters (Figure S5A), along which a distinct XCR1‐high subset emerged at the trajectory end (Figure 1H,I). Notably, XCR1 expression was markedly higher in dPMN‐MDSCs than in pPMN‐MDSCs, and the XCR1^+^PMN‐MDSC population identified in decidual CD45^+^ cells was distinct from that in peripheral blood. Crucially, this specific population was significantly reduced in the AMA group (Figure 1J–M). XCR1 is a classic marker for type 1 conventional dendritic cells (cDC1s) [36, 37]. By analyzing the expression of lineage‐defining markers in the scRNA‐seq data, we found that canonical dendritic cell (DC) markers were rarely expressed in the XCR1‐high neutrophil cluster_1 (Figure 1N; Figure S5B). Flow cytometry further confirmed low XCR1 expression in decidual DCs, T cells, natural killer (NK) cells, macrophages, and trophoblasts, consistent with levels in pPMN‐MDSCs (Figure S5C,D). Additionally, XCR1 expression in these cells remained unchanged between the HP and AMA groups (Figure S5E). These results demonstrate that a specific XCR1‐high dPMN‐MDSC subset is present at the maternal–fetal interface but is markedly decreased in AMA.

After identifying this XCR1‐high dPMN‐MDSC subset that was selectively diminished in AMA, we characterized the expression of its cognate ligands (XCL1 and XCL2) and determined their cellular source at the maternal–fetal interface. The results showed that both XCL1 and XCL2 were poorly expressed in dPMN‐MDSCs and remained unchanged between the HP and AMA groups (Figure S5F–H). At the maternal–fetal interface, dPMN‐MDSCs can be induced or activated by signals originating from trophoblasts [18]. RNA‐seq analysis of HLA‐G^+^ trophoblasts showed that, in addition to functional genes, *XCL1* (but not *XCL2*) was downregulated in trophoblasts from the AMA group (Figure S6A,B). These findings were further confirmed by qPCR, flow cytometry, and ELISA analyses (Figure 1O–Q). Crucially, XCL1 was primarily expressed in trophoblasts (Figure 1R), with flow cytometry further demonstrating high XCL1 expression in trophoblasts and negligible levels in DICs and DSCs (Figure S6C). These results confirm trophoblasts as the primary source of XCL1 at the maternal–fetal interface, with reduced production in AMA.

We next investigated the functional impact of impaired XCL1–XCR1 crosstalk on dPMN‐MDSCs. Specifically, we focused on GPFs that are essential for bone development and fetal growth, including OPN, OGN, PTN, vascular endothelial growth factor (VEGF), insulin‐like growth factor 2 (IGF2), and integrin alpha D (ITGAD) [38, 39, 40, 41]. The results showed that in the XCR1‐high neutrophil cluster_1, *SPP1* (encoding OPN), *OGN*, and *PTN* were significantly downregulated in AMA, whereas *VEGF*, *IGF2*, and *ITGAD* showed no intergroup differences (Figure S7A–F). These results were corroborated by qPCR and flow cytometry (Figure S7G,H). We then asked whether trophoblast‐derived XCL1 influenced the production of these GPFs in dPMN‐MDSCs. In co‐culture experiments, the GPF‐producing capacity of dPMN‐MDSCs was enhanced by supernatants collected from HP‐trophoblasts; however, this effect was diminished when cultured with the supernatants from AMA‐trophoblasts. Recombinant XCL1 (rXCL1) treatment effectively mimicked the enhancement observed under HP‐trophoblast stimulation. Moreover, the application of an anti‐XCR1‐neutralizing antibody (XCR1 mAb) successfully reversed the enhancement of GPF production induced by HP‐derived trophoblast stimulation. Importantly, regardless of the culture system used, GPF production remained lower in AMA‐derived dPMN‐MDSCs (Figure S7I–K). These data indicate a functional trophoblast–dPMN‐MDSC crosstalk driven by the XCL1–XCR1 axis; this axis promotes GPF secretion by dPMN‐MDSCs and supports fetal growth but is compromised in AMA.

### 2.2 Weakened Expression of the XCL1–XCR1 Axis Leads to FGR in AMA Mice by Diminishing dPMN‐MDSC Count and Function

**FIGURE 2:**
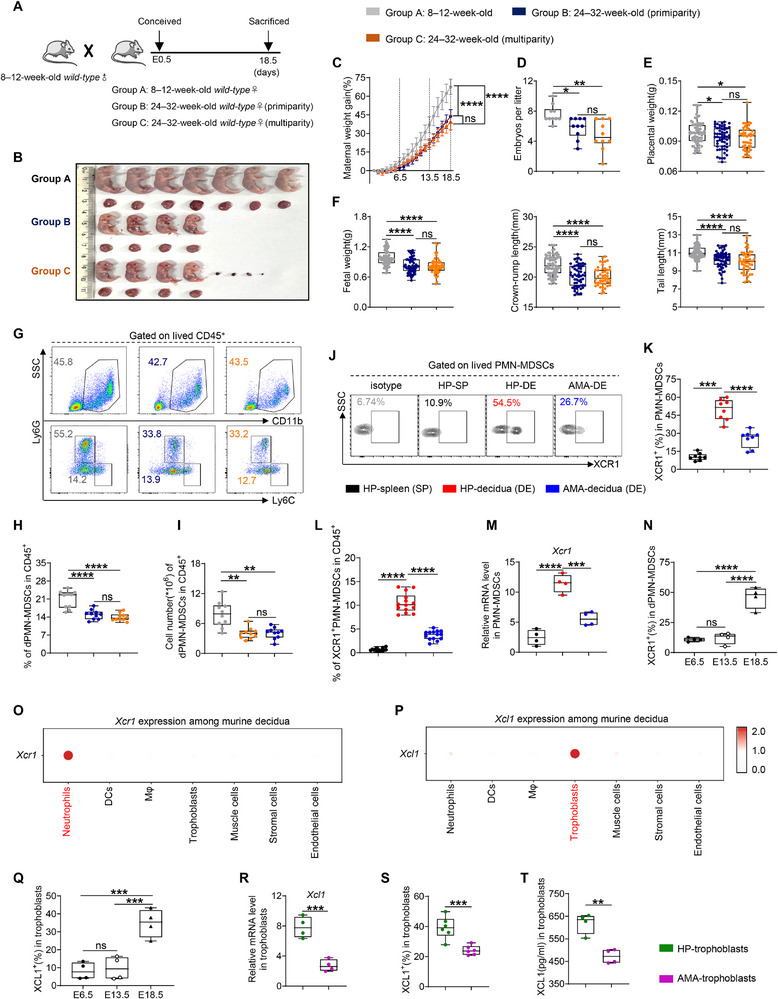
Weakened expression of the XCL1–XCR1 axis leads to FGR in AMA mice by diminishing dPMN‐MDSC count and function. (A) Cross strategies of different mouse models. (B–F) Representative image, maternal weight gain, embryo number per litter (*n =* 10), placental weight, and fetal indicators (*n =* 76, 58, and 48, respectively) in each group. (G–I) Representative flow cytometry and statistical analysis of dPMN‐MDSCs in CD45^+^ cells (*n =* 10). (J–L) Representative flow cytometry (J), percentages of XCR1^+^ cells in PMN‐MDSCs (K, *n =* 8), and statistical analysis of XCR1^+^PMN‐MDSCs (L, *n =* 14). (M) mRNA expression levels of *Xcr1* in PMN‐MDSCs (*n =* 4 replicates). (N) Percentages of XCR1^+^ cells in dPMN‐MDSCs across gestational stages (*n* = 4). (O) *Xcr1* expression among murine decidua at embryonic day E14. (P) *Xcl1* expression among murine decidua. (Q) Percentages of XCL1^+^ cells in trophoblasts across gestational stages (*n* = 4). (R–T) mRNA expression levels (R, *n* = 4 replicates), percentages (S, *n* = 6), and secretion levels (T, *n =* 4) of XCL1 in trophoblasts. Data are presented as mean ± SEM. Each dot represents a single mouse. ns, not significant; ^*^ *p* < 0.05, ^**^ *p* < 0.01, ^***^ *p* < 0.001, ^****^ *p* < 0.0001. Statistical significance was determined using a Student's *t‐*test (K, M, and R–T), Mann–Whitney test (K and L), or one‐way ANOVA (C–F, H, I, N, and Q). Post‐hoc analyses were performed using Tukey's test (C, E, F, H, N, and Q) or Dunnett's T3 test (D, F, and I).

To translate our clinical findings into an animal model and further examine the roles of dPMN‐MDSCs and the XCL1–XCR1 axis in AMA‐associated FGR, we established pregnant mouse cohorts based on maternal age and parity: young females (8–12 weeks, group A), aged primiparous females (24–32 weeks, group B), and aged multiparous females (24–32 weeks, group C)—all mated with young males (8–12 weeks) (Figure 2A). Consistent with the clinical observations of high FGR rates in women of AMA, the fetuses in groups B and C exhibited significant growth deficiencies, including reductions in maternal weight gain, embryo number per litter, placental and fetal weights, and crown‐to‐rump and tail lengths (Figure 2B–F). Simultaneously, the proportions of PMN‐MDSCs in the spleen (splenic PMN‐MDSCs) were lower in groups B and C than in group A, while no differences were observed in monocytic (M)‐MDSCs (Figure S8A–C). Since there were no notable differences in fetal growth markers or PMN‐MDSC levels between groups B and C, these groups were combined for subsequent experiments (Figure 2C–F; Figure S8B). Impairments in the activation of splenic PMN‐MDSCs were also observed in AMA mice, manifesting as weakened immunosuppressive activity and downregulated PMN‐MDSC‐related targets (Figure S8D–H). Notably, the reduction in PMN‐MDSC abundance was more prominent in the decidua (dPMN‐MDSCs) than in the spleen of AMA mice (Figure 2G–I; Figure S8B). However, no significant differences were observed in M‐MDSC levels in the decidua (dM‐MDSCs) (Figure S8I). In AMA mice, fetal sex did not affect dPMN‐MDSC levels or fetal growth parameters (Figure S9A–E). Furthermore, no significant exacerbation of inflammation was detected in the circulation or placental tissues of AMA mice (Figure S9F–H). These findings collectively indicate that pregnant AMA mice recapitulate human FGR phenotypes, with deficits specifically associated with reduced dPMN‐MDSC abundance and activation, independent of fetal sex or inflammation.

To clarify the role of the XCL1–XCR1 axis in these phenotypes, we analyzed XCR1 expression in the mouse model. In alignment with human results, dPMN‐MDSCs exhibited higher XCR1 expression than splenic PMN‐MDSCs, and a distinct XCR1^+^PMN‐MDSC population was identified among decidual CD45^+^ cells—with this subset significantly depleted in AMA mice (Figure 2J–M). Additionally, XCR1 expression in dPMN‐MDSCs exhibited temporal specificity, showing upregulation specifically during the third trimester, while remaining at relatively low levels throughout early and mid‐gestation (Figure 2N). Furthermore, no significant differences were observed between HP and AMA mice during these earlier gestational stages (Figure S10A). Reanalysis of scRNA‐seq data from murine decidual tissues reported by Jin et al., [42]. revealed that *Xcr1* was predominantly expressed in the neutrophil cluster, with minimal expression in other clusters (Figure 2O; Figure S10B–D). Flow cytometry analyses further confirmed low or absent XCR1 expression in trophoblasts and other DICs, including cDC1 (Figure S10E,F), and these levels remained unchanged between HP and AMA mice (Figure S10G). These findings demonstrate that XCR1 serves as a third‐trimester‐specific marker for murine dPMN‐MDSCs, with the corresponding cell subset diminished in AMA mice.

We also characterized XCL1 expression in the mouse model. Similar to human data, XCL1 expression in splenic and decidual PMN‐MDSCs did not differ between young and aged mice (Figure S10H). Instead, XCL1 was predominantly expressed in trophoblasts at the maternal–fetal interface, with negligible levels in DICs or DSCs (Figure 2P; Figure S11A). Notably, the expression pattern of XCL1 aligned with that of XCR1 (Figure 2Q), revealing that XCL1 was highly expressed in trophoblasts during the third trimester but significantly decreased in AMA mice (Figures 2R–T). In contrast, during early to mid‐gestational stages, XCL1 expression in trophoblasts remained relatively low, with no changes under AMA conditions (Figure S11B). These results indicate that trophoblasts are the primary source of XCL1 and that its third‐trimester‐specific expression is closely correlated with XCR1 in dPMN‐MDSCs; however, this pattern is disrupted in AMA mice.

We next investigated GPF expression in murine dPMN‐MDSCs and their regulation by the XCL1–XCR1 axis. First, we assessed the temporal and cohort‐specific expression of OPN, OGN, and PTN. Consistent with the gestational specificity of the XCL1–XCR1 axis, the production of these three GPFs by dPMN‐MDSCs was upregulated during the third trimester (Figure S12A) but significantly impaired in AMA mice (Figure S12B,C). In contrast, GPF production remained relatively low during early to mid‐gestation, with no differences between young and aged mice (Figure S12D–F). To directly validate the regulatory role of the XCL1–XCR1 axis in GPF secretion, we replicated the human co‐culture experiments in the mouse model. Compared to the culture of dPMN‐MDSCs alone, GPF secretion was increased under stimulation by trophoblast‐cultured supernatant; however, this effect was significantly weaker with supernatants from AMA‐derived trophoblasts. rXCL1 treatment enhanced GPF production, mimicking the effect of the HP trophoblast supernatant. Furthermore, XCR1 mAb effectively eliminated this trophoblast stimulus. Notably, GPF production remained consistently lower in AMA mice across all employed culture systems (Figure S12G–I). Collectively, these findings establish the XCL1–XCR1 axis as an evolutionarily conserved regulator of GPF secretion by dPMN‐MDSCs; its dysfunction, resulting from diminished trophoblast‐derived XCL1, directly compromises the GPF secretory capacity of these cells under AMA conditions.

### 2.3 Decidual XCR1+PMN‐MDSCs are Essential for Fetal Development

**FIGURE 3:**
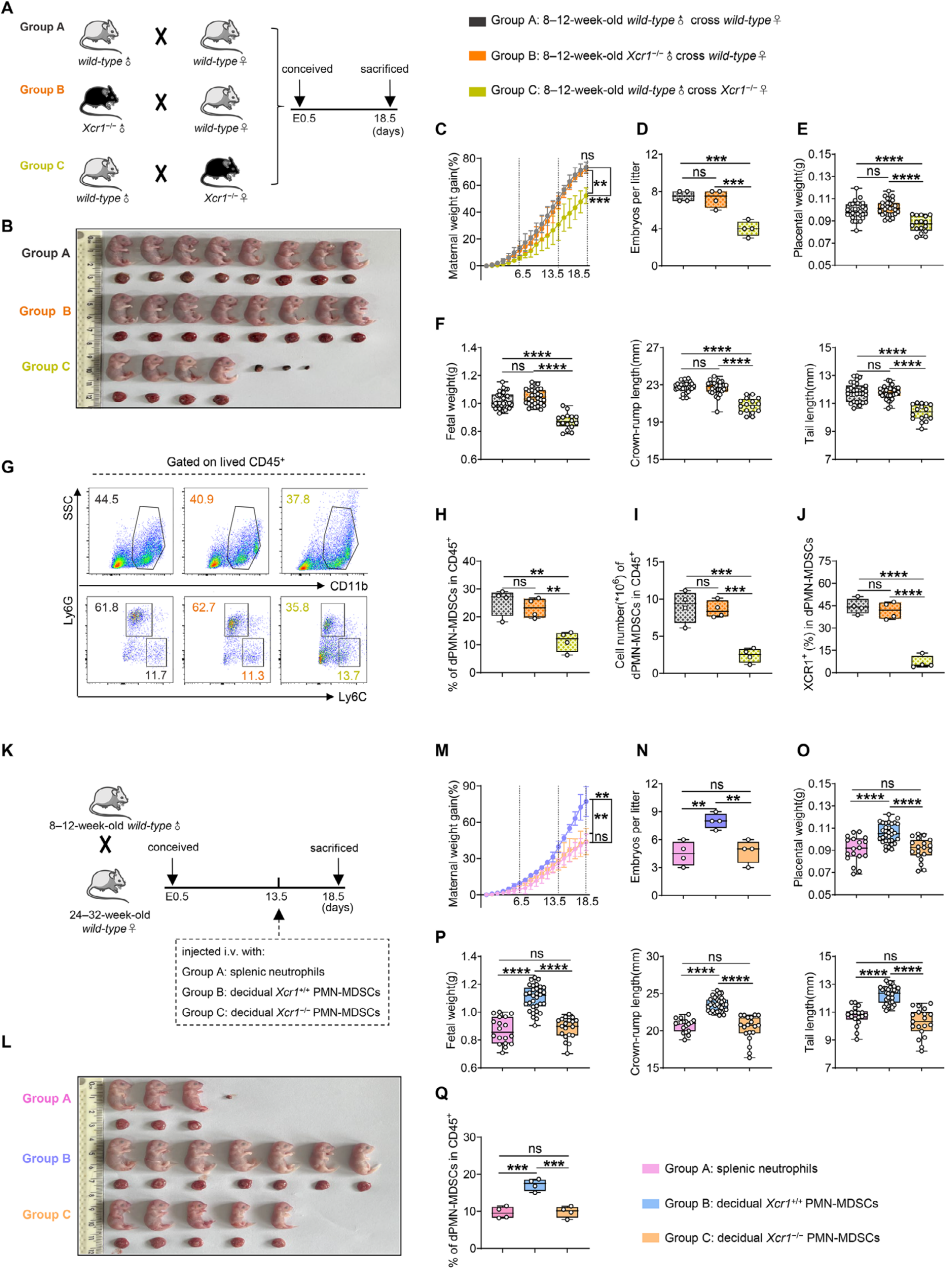
Decidual XCR1^+^PMN‐MDSCs are essential for fetal development. (A) Cross strategies of 8–12‐week‐old males and females with different genotype backgrounds. (B–F) Representative image, maternal weight gain, embryo number per litter (*n* = 4), placental weight, and fetal growth indicators (*n* = 30, 29, and 16, respectively) in different groups. (G–I) Representative flow cytometry and statistical analysis of dPMN‐MDSCs in CD45^+^ cells (*n* = 4). (J) Percentages of XCR1^+^ cells in dPMN‐MDSCs (*n* = 4). (K) 24–32‐week‐old AMA mice were injected with splenic neutrophils from non‐pregnant wild‐type mice, dPMN‐MDSCs from pregnant *Xcr1* ^+/+^ or *Xcr1* ^−/−^ mice intravenously (i.v.) at E13.5. (L–P) Representative image, maternal weight gain, embryo number per litter (*n* = 4), placental weight, and fetal growth indicators (*n* = 18, 32, and 19, respectively) across the groups. (Q) Proportions of dPMN‐MDSCs in CD45^+^ cells (*n* = 4). Data are presented as mean ± SEM. Each dot represents a single mouse. ns, not significant; ^*^ *p* < 0.05, ^**^ *p* < 0.01, ^***^ *p* < 0.001, ^****^ *p* < 0.0001. Statistical significance was determined using one‐way ANOVA (C–F, H–J, and M–Q). Post‐hoc analyses were performed using Tukey's test (C–F, H–J, and M–Q) or Dunnett's T3 test (P).

We sought to directly confirm the importance of decidual XCR1^+^PMN‐MDSCs for fetal development using genetic knockout and cell adoptive transfer experiments. First, multiple crossing strategies involving wild‐type and *Xcr1^−/−^* background mice were designed (Figure 3A). Compared to the control group (group A), paternal *Xcr1^−/−^* did not induce FGR phenotypes, whereas maternal *Xcr1^−/−^* exhibited spontaneous FGR (Figure 3B–F). The abundance of dPMN‐MDSCs was significantly reduced in the maternal *Xcr1^−/−^* group, with no observable changes in the paternal *Xcr1^−/−^* group (Figure 3G–I). However, maternal *Xcr1^−/−^* did not influence the levels of dM‐MDSCs, splenic PMN‐MDSCs, splenic M‐MDSCs, or other DICs (Figure S13A–D). Moreover, maternal *Xcr1^−/−^* did not exacerbate inflammation in either the circulation or placental tissues (Figure S13E–G). Notably, XCR1 expression was significantly decreased specifically within dPMN‐MDSCs of the maternal *Xcr1^−/−^* group, as opposed to other DICs (Figure 3J; Figure S13H). These results demonstrate that maternal (but not paternal) *Xcr1* deficiency drives spontaneous FGR, which is directly caused by the loss of decidual XCR1^+^PMN‐MDSCs, independent of other MDSC subsets, DICs, or inflammation.

To further validate that decidual XCR1^+^PMN‐MDSCs are essential for rescuing FGR, we performed adoptive transfer experiments. Specifically, AMA mice were intravenously injected at embryonic day (E) 13.5 with one of three cell populations: splenic neutrophils from non‐pregnant wild‐type mice (group A), dPMN‐MDSCs from pregnant *Xcr1^+^/^+^* mice (group B), or dPMN‐MDSCs from pregnant *Xcr1^−/−^* mice (group C) (Figure 3K). Strikingly, AMA mice receiving XCR1‐expressing dPMN‐MDSCs (group B) exhibited improved fetal development, including increases in maternal weight gain, embryo number per litter, placental and fetal weights, as well as crown‐to‐rump and tail lengths (Figure 3L–P). This improvement was associated with a specific increase in dPMN‐MDSC levels (Figure 3Q), without changes in dM‐MDSCs, splenic PMN‐MDSCs, or splenic M‐MDSCs (Figure S14A–C). In contrast, no improvements in fetal development or alterations in MDSC subsets were observed for the XCR1‐deficient groups (groups A and C) (Figure 3L–Q; Figure S14A–C). Together, these results establish that the adoptive transfer of decidual XCR1^+^PMN‐MDSCs specifically ameliorates FGR phenotypes in AMA mice, underscoring their essential role in fetal development.

### 2.4 XCL1 Effectively Improves FGR Phenotypes in AMA Mice but Not in Pregnant Xcr1−/− Mice

**FIGURE 4:**
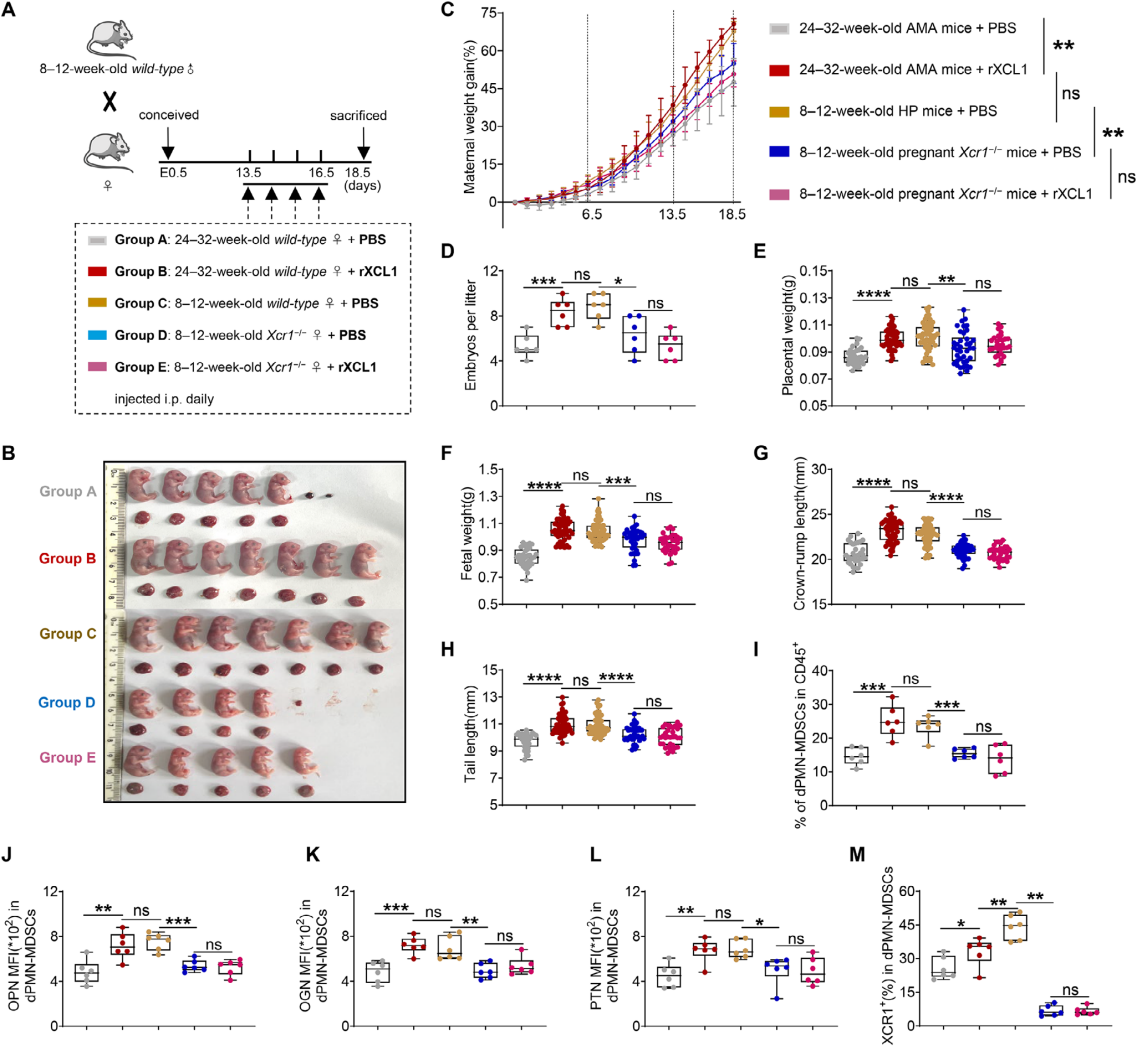
XCL1 effectively improves FGR phenotypes in AMA mice but not in pregnant *Xcr1^−/−^* mice. (A) 24–32‐week‐old AMA and 8–12‐week‐old pregnant *Xcr1^−/−^* mice were injected daily with PBS or 0.2 µg recombinant mouse XCL1 protein (rXCL1) intraperitoneally (i.p.) from E13.5 to E16.5. (B–H) Representative image, maternal weight gain, embryo number per litter (*n* = 6), placental weight, and fetal growth indicators (*n* = 32, 50, 53, 38, and 32, respectively) between the groups. (I) Proportions of dPMN‐MDSCs in CD45^+^ cells (*n =* 6). (J–L) Mean fluorescence intensity (MFI) of osteopontin (OPN), osteoglycin (OGN), and pleiotrophin (PTN) in dPMN‐MDSCs (*n =* 6). (M) Percentages of XCR1^+^ cells in dPMN‐MDSCs (*n =* 6). Data are presented as mean ± SEM. Each dot represents a single mouse. ns, not significant; ^*^ *p* < 0.05, ^**^ *p* < 0.01, ^***^ *p* < 0.001, ^****^ *p* < 0.0001. Statistical significance was determined using a Student's *t‐*test (C–M) or Mann–Whitney test (C, E, G, I, and M).

Having confirmed that decidual XCR1^+^PMN‐MDSCs are indispensable for fetal development, we next asked whether supplementing their cognate ligand, XCL1, could rescue FGR. We performed XCL1 supplementation experiments in two FGR models: 24–32‐week‐old AMA mice and 8–12‐week‐old pregnant *Xcr1^−/−^* mice. In both models, mice were intraperitoneally administered rXCL1 or PBS daily from E13.5 to E16.5, with PBS‐treated HP mice serving as the control group (Figure 4A). rXCL1 injection significantly rescued FGR phenotypes in AMA mice, displaying improved maternal weight gain and embryo number per litter, increased placental and fetal weights, and enhanced crown‐to‐rump and tail lengths (Figure 4B–H); these parameters approached those of the PBS‐treated HP group. Following rXCL1 supplementation, the abundance and function of dPMN‐MDSCs were increased in AMA mice, showing no significant differences relative to the PBS‐treated HP group (Figure 4I–L). In contrast, no such improvements or changes were observed in pregnant *Xcr1^−/−^* mice treated with rXCL1 (Figure 4B–L). Additionally, rXCL1 administration did not affect dM‐MDSC, splenic PMN‐MDSC, or splenic M‐MDSC levels in either AMA or pregnant *Xcr1^−/−^* mice (Figure S14D–F). Notably, rXCL1 administration restored XCR1 expression in dPMN‐MDSCs of AMA mice but not in pregnant *Xcr1^−/−^* mice (Figure 4M); this treatment barely changed intracellular XCL1 expression in trophoblasts of both AMA and pregnant *Xcr1^−/−^* mice (Figure S14G). These findings demonstrate that exogenous XCL1 can rescue FGR and dPMN‐MDSC dysfunction in AMA mice—provided that there is functional XCR1 in dPMN‐MDSCs—establishing the specific role of the XCL1–XCR1 axis in fetal growth.

### 2.5 Attenuated XCL1–XCR1 Expression that Causes FGR is Related to Impaired FOXO1 Expression and Activity

**FIGURE 5:**
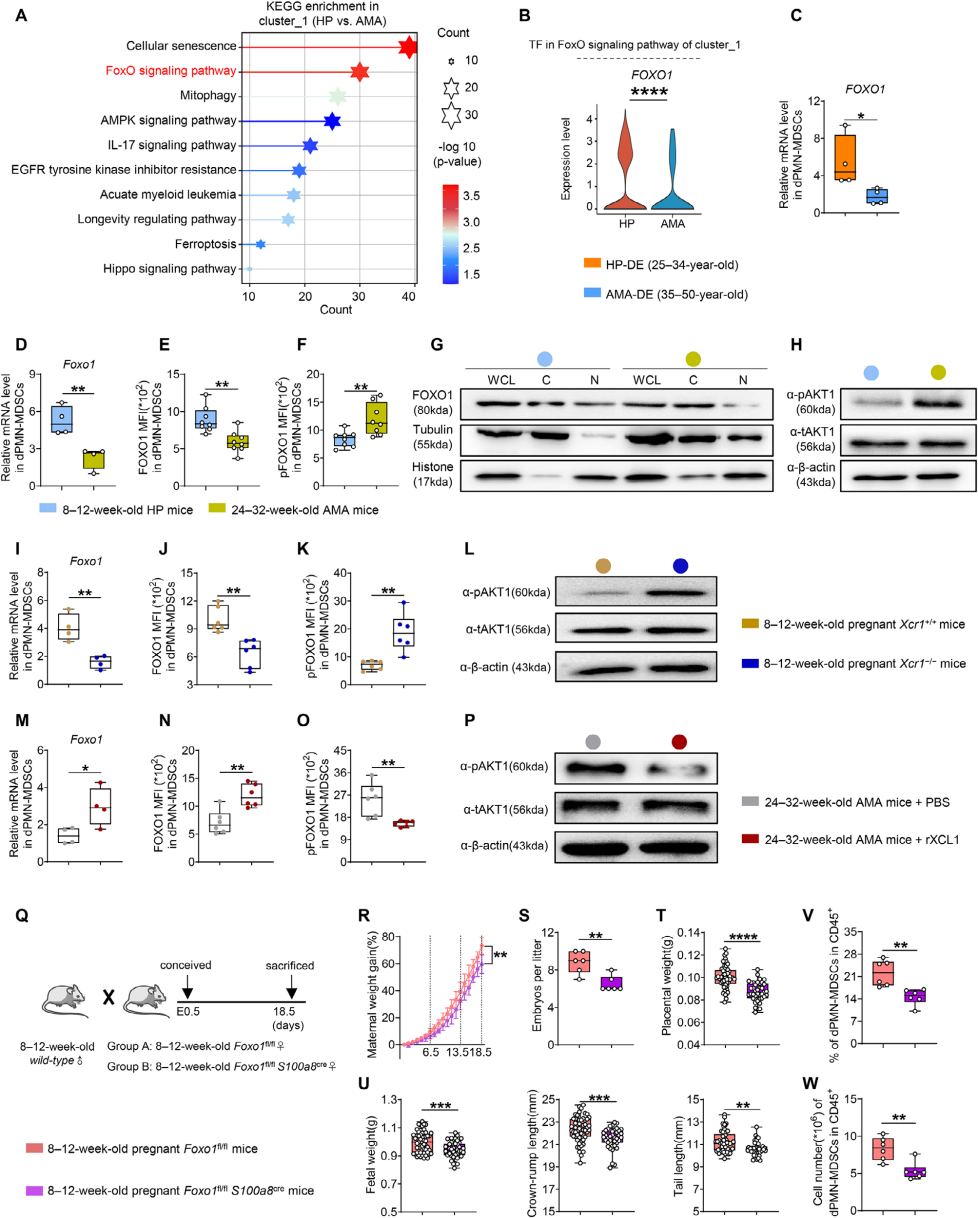
Attenuated XCL1–XCR1 expression that causes FGR is related to impaired FOXO1 expression and activity. (A, B) Enriched signaling pathways of DEGs in neutrophil cluster_1 between the HP and AMA groups (A) and the transcriptional factor in the FoxO signaling pathway (B). (C) mRNA expression level of *FOXO1* in human dPMN‐MDSCs (*n =* 4 replicates). (D–F) mRNA expression level (D, *n =* 4 replicates) and MFI of FOXO1 (E, *n =* 8) and phosphorylated FOXO1 (pFOXO1) (F, *n =* 8) in dPMN‐MDSCs. (G) Western blot analysis of whole‐cell lysate (WCL), cytoplasmic (C), and nuclear (N) distributions of FOXO1 expression in dPMN‐MDSCs of HP and AMA mice (replicated twice). (H) Western blot analysis of phosphorylated (p)‐ and total (t)‐AKT1 in dPMN‐MDSCs (replicated three times). (I–K, M–O) mRNA expression levels (I, M, *n =* 4 replicates) and MFI of FOXO1 (J, N, *n =* 6) and pFOXO1 (K, O, *n =* 6) in dPMN‐MDSCs. (L, P) Western blot analysis of p‐ and t‐AKT1 in dPMN‐MDSCs (replicated three times). (Q) 8–12‐week‐old *Foxo1* ^fl/fl^ and *Foxo1* ^fl/fl^ *S100a8* ^cre^ females mated with 8–12‐week‐old males. (R–U) Maternal weight gain, embryo number per litter (*n* = 6), placental weight, and fetal growth indicators (*n* = 53 and 39, respectively) between the groups. (V, W) Statistical analysis of dPMN‐MDSCs in CD45^+^ cells (*n* = 6). Data are presented as mean ± SEM. Each dot represents a single individual or mouse. ns, not significant; ^*^ *p* < 0.05, ^**^ *p* < 0.01, ^***^ *p* < 0.001, ^****^ *p* < 0.0001. Statistical significance was determined using a Student's *t*‐test (C–F, I, J, M, N, and R–W), Mann–Whitney test (K and O), or Wilcoxon test (B).

After confirming that the XCL1‐mediated rescue of FGR depends on decidual XCR1^+^PMN‐MDSCs, we next explored the downstream molecular mechanism linking this axis to dPMN‐MDSC function, specifically focusing on transcription factor activity and signaling cascades. KEGG pathway analysis of neutrophil cluster_1 revealed significant enrichment of the FoxO signaling pathway in differentially expressed genes (DEGs) (Figure 5A). Among FoxO family transcription factors, only FOXO1 was significantly downregulated in dPMN‐MDSCs of AMA individuals, which was validated by qPCR (Figure 5B,C; Figure S15A). Post‐translational modifications of FOXO1, such as phosphorylation regulated by AKT1, alter its subcellular localization and activity [43]. Both qPCR and flow cytometry results revealed decreased FOXO1 and increased pFOXO1 levels in dPMN‐MDSCs of AMA mice (Figure 5D–F). Based on these findings, the nucleocytoplasmic distribution of FOXO1 was investigated. FOXO1 was primarily located in the nuclear extracts of HP‐derived dPMN‐MDSCs, with a small proportion in the cytoplasmic fraction; this pattern was reversed in the AMA group (Figure 5G). Consistently, the level of pAKT1 in dPMN‐MDSCs of AMA mice increased (Figure 5H). These experiments were also performed in pregnant *Xcr1* ^+/+^ and *Xcr1^−/−^* mice, with consistent results between pregnant *Xcr1^−/−^* and AMA mice, revealing a reduction in FOXO1 expression alongside elevated levels of pFOXO1 and pAKT1 in dPMN‐MDSCs (Figure 5I–L). Notably, AMA mice treated with rXCL1 exhibited significant restoration of FOXO1 expression and effective inhibition of pFOXO1 and pAKT1 levels in dPMN‐MDSCs compared to those administered PBS (Figure 5M–P). FOXO1 expression and activity also showed temporal specificity, revealing a significant increase in the third trimester while remaining at low levels during early to mid‐gestation. Notably, pFOXO1 and pAKT1 levels exhibited an inverse pattern (Figure S15B). However, no changes were observed in this axis between HP and AMA mice at the earliest time point (Figure S15C–E). These data confirm a stage‐specific regulatory role of the XCL1–XCR1 axis in dPMN‐MDSCs, by which it inhibits AKT1 and enhances FOXO1 expression**—**a mechanism disrupted in both AMA and pregnant *Xcr1^−/−^* mice.

Maternal neutrophil‐specific *Foxo1*‐deficient mice (*Foxo1* ^fl/fl^ *S100a8* ^cre^) were next used to validate the critical role of FOXO1 (Figure 5Q). Consistent with the fetal observations in AMA and pregnant *Xcr1^−/−^* mice, pregnant *Foxo1* ^fl/fl^ *S100a8* ^cre^ mice displayed developmental delays and poor fetal growth (Figure 5R–U; Figure S16A). The abundance of dPMN‐MDSCs was reduced in pregnant *Foxo1* ^fl/fl^ *S100a8* ^cre^ mice (Figure 5V,W); however, no significant differences were observed in the levels of dM‐MDSCs, splenic PMN‐MDSCs, or splenic M‐MDSCs (Figure S16B–F). Moreover, *Foxo1* deficiency did not influence either XCR1 or pAKT1 levels in dPMN‐MDSCs (Figure S16G–J), nor did it affect XCL1 expression in trophoblasts (Figure S16K,L). Additionally, both mRNA and protein analyses confirmed the successful deletion of FOXO1 in dPMN‐MDSCs (Figure S16M,N). In summary, the XCL1–XCR1 axis enhances FOXO1 activity in dPMN‐MDSCs by suppressing AKT1‐mediated phosphorylation. Critically, neutrophil‐specific *Foxo1* deficiency recapitulates the FGR phenotypes of AMA or pregnant *Xcr1^−/−^* mice, confirming FOXO1 as a key downstream effector of this signaling axis.

### 2.6 Activated FOXO1 Promotes the Transcription of OXPHOS‐Related Targets in Decidual XCR1+PMN‐MDSCs

**FIGURE 6:**
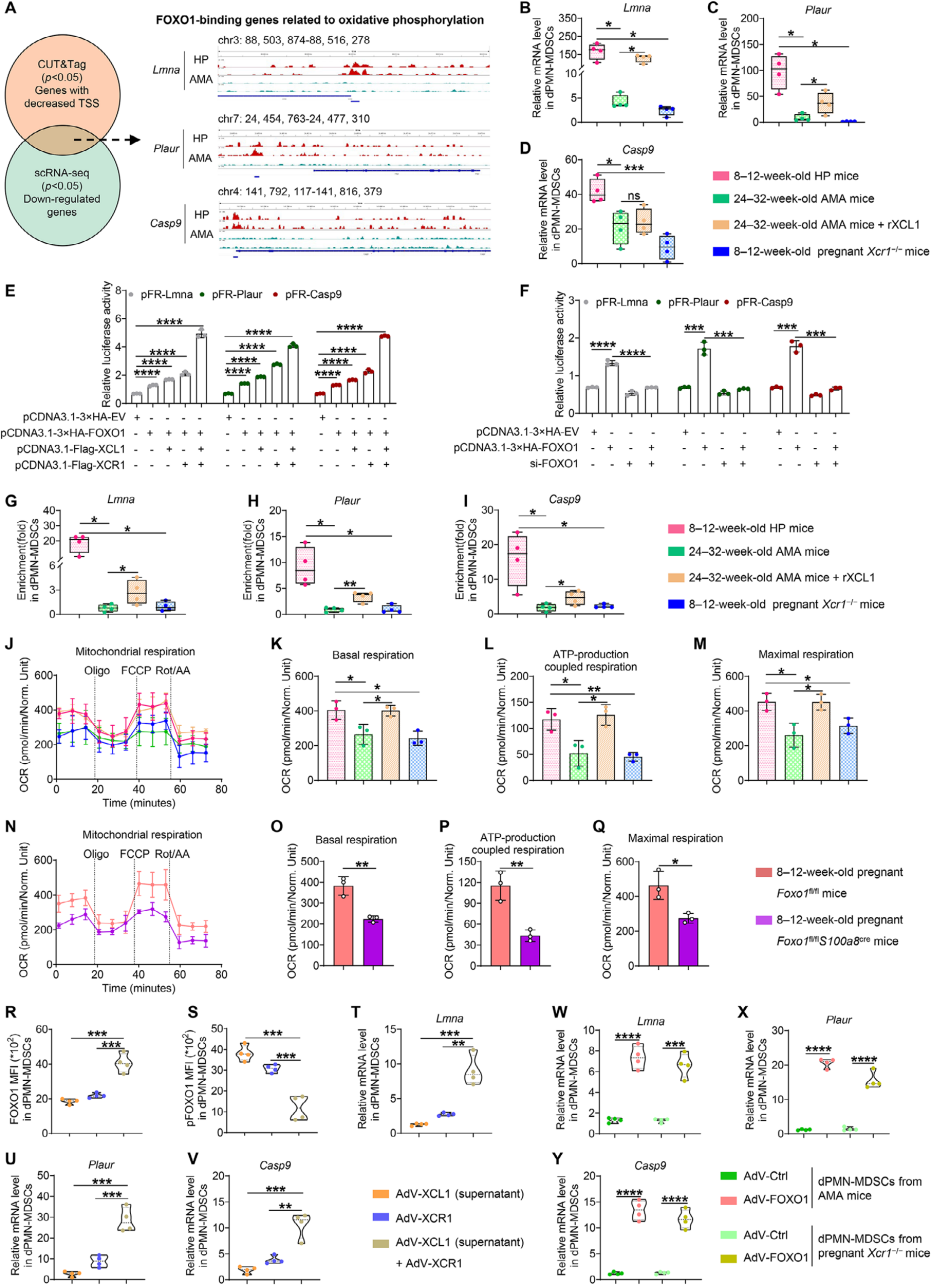
Activated FOXO1 promotes the transcription of OXPHOS‐related targets in decidual XCR1^+^PMN‐MDSCs. (A) Venn diagram showing FOXO1 downstream target selection and IGV visualization showing the target binding peaks. (B–D) mRNA expression levels of *Lnma*, *Plaur*, and *Casp9* in decidual XCR1^+^PMN‐MDSCs across groups (*n =* 4 replicates). (E) Dual‐luciferase reporter assay after transfection with the indicated plasmids (*n* = 3 replicates). (F) Relative luciferase activity of the *Lmna*, *Plaur*, and *Casp9* promoters upon FOXO1 overexpression or knockdown (*n* = 3 replicates). (G–I) Enrichment of FOXO1 in the target promoters of decidual XCR1^+^PMN‐MDSCs across groups (*n =* 4 replicates). (J–M) Basal oxygen consumption rate (OCR), ATP production, and maximal respiratory capacity of decidual XCR1^+^PMN‐MDSCs across groups (*n* = 3 replicates). (N–Q) Basal OCR, ATP production, and maximal respiratory capacity of decidual XCR1^+^PMN‐MDSCs from pregnant *Foxo1* ^fl/fl^ and *Foxo1* ^fl/fl^ *S100a8* ^cre^ mice (*n* = 3 replicates). (R, S) MFI of FOXO1 and pFOXO1 in dPMN‐MDSCs from AMA mice after treatment with adenoviral vector (AdV)‐XCL1 conditioned supernatant, transfection with AdV‐XCR1, or both (*n* = 4). (T–V) mRNA expression levels of *Lmna*, *Plaur*, and *Casp9* in dPMN‐MDSCs under different treatments (*n* = 4 replicates). (W–Y) mRNA expression levels of *Lmna*, *Plaur*, and *Casp9* in dPMN‐MDSCs from AMA and pregnant *Xcr1* ^–/–^ mice after transfection with either AdV‐control (AdV‐Ctrl) or AdV‐FOXO1 (*n* = 4 replicates). Data are presented as mean ± SEM. Each dot represents a single mouse. ns, not significant; ^*^ *p* < 0.05, ^**^ *p* < 0.01, ^***^ *p* < 0.001, ^****^ *p* < 0.0001. Statistical significance was determined using a Student's *t‐*test (C–I, K–M, and O–Y) or Mann–Whitney test (B, C, and G–I).

Building on the above findings that the XCL1–XCR1–FOXO1 axis is specific to late gestation and regulates dPMN‐MDSC function, we next aimed to identify the downstream transcriptional targets of FOXO1. We performed cleavage under targets and tagmentation (CUT&Tag) sequencing with an anti‐FOXO1 antibody on sorted decidual XCR1^+^PMN‐MDSCs isolated from HP and AMA mice, the latter representing a model in which this axis is impaired (Figure S17A). Analysis of transcriptional start sites (TSSs) revealed a reduction in enriched FOXO1‐binding peaks in decidual XCR1^+^PMN‐MDSCs from the AMA group, with a notable decrease in promoter region (54.5% in HP vs. 39.5% in AMA; Figure S17B,C). Gene Ontology (GO) analysis associated the differentially targeted genes between the HP and AMA groups with mitochondrial processes (Figure S17D). Crucially, integrating the CUT&Tag and scRNA‐seq data identified three OXPHOS‐related genes—*Lmna*, *Plaur*, and *Casp9*—that exhibited significantly reduced FOXO1‐binding peaks in their promoter regions in AMA mice (Figure 6A). Subsequent qPCR validation confirmed the downregulation of these genes in decidual XCR1^+^PMN‐MDSCs from both AMA and pregnant *Xcr1^−/−^* mice, while rXCL1 administration restored their expression in AMA mice (Figure 6B–D). Consistent with the late‐gestational specificity of the XCL1–XCR1–FOXO1 axis, the expression levels of *Lmna*, *Plaur*, and *Casp9* were significantly higher during the third trimester compared to early‐to‐mid gestation, with no significant differences observed between HP and AMA mice at earlier time points (Figure S17E–H). These results indicate that the XCL1–XCR1–FOXO1 axis specifically regulates the transcription of OXPHOS‐related genes in third‐trimester decidual XCR1^+^PMN‐MDSCs; conversely, impairment in this axis, as seen in AMA or pregnant *Xcr1^−/−^* mice, reduces their expression.

To confirm the direct binding of FOXO1 to these targets and their subsequent activation, we performed dual‐luciferase assays using promoter reporter plasmids for *Lmna*, *Plaur*, and *Casp9*. Western blot analysis confirmed successful FOXO1, XCL1, and XCR1 overexpression in 293T cells (Figure S18A). FOXO1 overexpression alone increased luciferase activity for all three promoters, and this activation was further amplified by the co‐overexpression of either XCL1 or XCR1, with the strongest effect observed when all three factors were combined (Figure 6E). In contrast, siRNA‐mediated knockdown of FOXO1 (si‐FOXO1) markedly reduced both FOXO1 protein levels and promoter activities relative to FOXO1 overexpression (Figure 6F; Figure S18B). To evaluate FOXO1 binding in vivo, chromatin immunoprecipitation was performed followed by quantitative PCR (ChIP‐qPCR) at the FOXO1‐binding motifs (Figure S18C,D). Consistent with the reporter assays, FOXO1 enrichment at the *Lmna*, *Plaur*, and *Casp9* promoters was reduced in decidual XCR1^+^PMN‐MDSCs from AMA and pregnant *Xcr1* ^−/−^ mice (Figure 6G–I). Notably, rXCL1 administration substantially restored FOXO1 binding in AMA mice (Figure 6G–I). Collectively, these findings demonstrate that FOXO1 directly binds to the promoters of *Lmna*, *Plaur*, and *Casp9* to drive their transcriptional activation, and that this regulation is enhanced by XCL1–XCR1 signaling.

Given the downregulation of OXPHOS‐related targets in both AMA and pregnant *Xcr1^−/−^* mice, we next investigated whether impaired XCL1–XCR1–FOXO1 signaling contributes to metabolic alterations in decidual XCR1^+^PMN‐MDSCs. Flow cytometry‐based analysis of single‐cell metabolism by profiling translation inhibition (SCENITH) revealed reduced mitochondrial dependency, increased glucose and glycolytic dependency, diminished ATP production, and elevated lactate levels in decidual XCR1^+^PMN‐MDSCs of AMA and pregnant *Xcr1^−/−^* mice (Figure S19A–D). Notably, these metabolic deficits were reversed by rXCL1 treatment in AMA mice (Figure S19E,F). Seahorse assays further confirmed these metabolic changes, demonstrating that basal and maximal oxygen consumption rates (OCR), as well as ATP‐linked respiration, were significantly reduced (Figure 6J–M), whereas basal glycolysis and glycolytic capacity—measured as extracellular acidification rate (ECAR)—were enhanced in decidual XCR1^+^PMN‐MDSCs from AMA and pregnant *Xcr1* ^−/−^ mice (Figure S19G–I). Importantly, rXCL1 administration effectively restored normal metabolic function (Figure 6J–M; Figure S19G–I).

Next, to directly establish the role of FOXO1 in mediating these metabolic phenotypes, we employed pregnant *Foxo1* ^fl/fl^ *S100a8* ^cre^ mice, which exhibit dPMN‐MDSC‐specific deletion of FOXO1. Decidual XCR1^+^PMN‐MDSCs from these animals displayed reduced mRNA expression levels of *Lmna*, *Plaur*, and *Casp9* (Figure S20A) and exhibited a pronounced metabolic imbalance characterized by decreased mitochondrial activity and enhanced glycolysis (Figure 6N–Q; Figure S20B–F). These data indicate that the XCL1–XCR1–FOXO1 axis controls metabolic reprogramming toward OXPHOS in decidual XCR1^+^PMN‐MDSCs, with axis impairment or FOXO1 deletion triggering a compensatory glycolytic program.

Finally, to confirm the causal role of the XCL1–XCR1 axis in FOXO1‐driven OXPHOS, we constructed adenoviral vectors overexpressing XCL1 (AdV‐XCL1), XCR1 (AdV‐XCR1), and FOXO1 (AdV‐FOXO1). Successful transfection and expression in trophoblasts (AdV‐XCL1; Figure S21A–E) and dPMN‐MDSCs (AdV‐XCR1 and AdV‐FOXO1; Figure S21F–M) were validated at the mRNA and protein levels. Treating dPMN‐MDSCs from AMA mice with conditioned supernatant from AdV‐XCL1‐transduced trophoblasts in combination with AdV‐XCR1 transfection significantly increased total FOXO1 expression, reduced pFOXO1 levels (Figure 6R,S), and upregulated *Lmna*, *Plaur*, and *Casp9* levels compared to either treatment alone (Figure 6T–V). Similarly, the transduction of dPMN‐MDSCs from either AMA or pregnant *Xcr1* ^−/−^ mice with AdV‐FOXO1 increased the expression of these OXPHOS‐related target genes relative to control vector (Figure 6W–Y). Collectively, these gain‐of‐function experiments demonstrate that restoring XCL1, XCR1, or FOXO1 expression enhances the transcription of OXPHOS‐related targets in dPMN‐MDSCs with impaired axis signaling, confirming the causal role of the XCL1–XCR1–FOXO1 axis in metabolic regulation.

### 2.7 OXPHOS Activation Rescues Developmental Delays Associated With Impaired Decidual XCR1+PMN‐MDSCs

**FIGURE 7:**
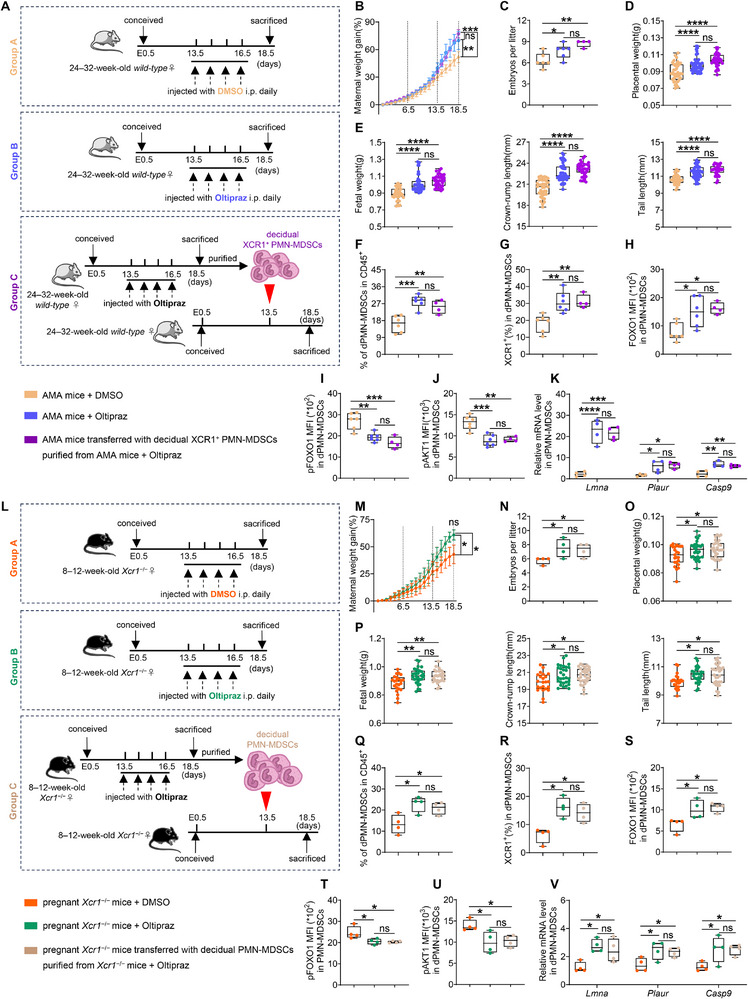
OXPHOS activation rescues developmental delays associated with impaired decidual XCR1^+^PMN‐MDSCs. (A) 24–32‐week‐old AMA mice were subjected to the following treatments: daily injection with the control vehicle or 2.0 mg/kg Oltipraz from E13.5 to E16.5 or the transfer of decidual XCR1^+^PMN‐MDSCs isolated from Oltipraz‐treated AMA donors at E13.5. (B–E) Maternal weight gain (B, *n* = 6, 6, and 4, respectively), embryo number per litter (C, *n* = 6, 6, and 4, respectively), and fetal growth indicators (D, E, *n* = 38, 46, and 35, respectively) in the respective indicated groups. (F) Percentages of dPMN‐MDSCs in CD45^+^ cells (*n* = 6, 6, and 4, respectively). (G) Percentages of XCR1^+^ cells in dPMN‐MDSCs (*n* = 6, 6, and 4, respectively). (H–J) MFI of FOXO1 (H), pFOXO1 (I), and pAKT1 (J) in dPMN‐MDSCs (*n* = 6, 6, and 4, respectively). (K) mRNA expression levels of *Lmna*, *Plaur*, and *Casp9* in dPMN‐MDSCs (*n =* 4 replicates). (L) 8–12‐week‐old pregnant *Xcr1^−/−^* mice were subjected to the following treatments: daily injection with the control vehicle or 2.0 mg/kg Oltipraz from E13.5 to E16.5 or the transfer of dPMN‐MDSCs isolated from Oltipraz‐treated pregnant *Xcr1* ^−/−^ donors at E13.5. (M–P) Maternal weight gain (M, *n* = 4), embryo number per litter (N, *n* = 4), and fetal growth parameters (O, P, *n* = 23, 30, and 29, respectively). (Q) Percentages of dPMN‐MDSCs in CD45^+^ cells (*n* = 4). (R) Percentages of XCR1^+^ cells in dPMN‐MDSCs (*n* = 4). S–U) MFI of FOXO1 (S), pFOXO1 (T), and pAKT1 (U) in dPMN‐MDSCs (*n* = 4). V) mRNA expression levels of *Lmna*, *Plaur*, and *Casp9* in dPMN‐MDSCs (*n =* 4 replicates). Data are presented as mean ± SEM. Each dot represents a single mouse. ns, not significant; ^*^ *p* < 0.05, ^**^ *p* < 0.01, ^***^ *p* < 0.001, ^****^ *p* < 0.0001. Statistical significance was determined using one‐way ANOVA (B–K and M–V). Post‐hoc analyses were performed using Tukey's test (B–K, O–Q, and S–V), Dunn's test (E and N), or Dunnett's T3 test (M and R).

Given the crucial role of the XCL1–XCR1–FOXO1 axis in metabolic regulation, we next tested whether activating OXPHOS in decidual XCR1^+^PMN‐MDSCs could rescue FGR in AMA mice and whether this effect was XCR1‐dependent. AMA mice received one of three treatments: intraperitoneal administration of either Oltipraz (an OXPHOS activator that operates through the nuclear factor erythroid 2‐related factor pathway) [44, 45] or a control vehicle, or adoptive transfer of decidual XCR1^+^PMN‐MDSCs isolated from Oltipraz‐treated AMA mice (Figure 7A). The developmental delays in AMA mice were significantly reversed following Oltipraz treatment or cell transfer (Figure 7B–E). Both interventions specifically increased the levels of dPMN‐MDSCs (Figure 7F) but not dM‐MDSCs, splenic PMN‐MDSCs, splenic M‐MDSCs, or other DICs (Figure S22A–D). These treatments also increased XCR1 expression in dPMN‐MDSCs (Figure 7G) but not in other DICs (Figure S22E). Notably, Oltipraz treatment and cell transfer restored FOXO1 expression (Figure 7H; Figure S22F), reduced pFOXO1 and pAKT1 levels (Figure 7I,J), upregulated OXPHOS‐related targets (Figure 7K), and enhanced mitochondrial activity (Figure S22G,H) in decidual XCR1^+^PMN‐MDSCs. Crucially, no significant differences were observed between direct Oltipraz treatment and the transfer of Oltipraz‐derived decidual XCR1^+^PMN‐MDSCs (Figure 7B–K; Figure S22A–H). These findings confirm that Oltipraz‐driven fetal growth recovery is specifically mediated by activating the XCR1–FOXO1–OXPHOS signaling pathway in dPMN‐MDSCs.

To investigate the association between OXPHOS and nutrient handling, we evaluated the expression of nutrient transporters in dPMN‐MDSCs [46, 47, 48]. The results showed that the expression of fatty acid transport protein 1 (*Fatp1*) and 2 (*Fatp2*) was upregulated, while glucose transporter type 4 (*Glut4*) was downregulated in decidual XCR1^+^PMN‐MDSCs following Oltipraz treatment or cell transfer (Figure S22I). Moreover, these treatments did not change XCL1 expression in trophoblasts (Figure S22J,K). Thus, these data indicate that OXPHOS activation in decidual XCR1^+^PMN‐MDSCs alters their nutrient transporter profile, suggesting a potential adaptation in nutrient handling.

To confirm the XCR1 dependence of FGR rescue, we repeated these experiments in pregnant *Xcr1* ^–^ *^/^* ^–^ mice (Figure 7L). Although fetal growth markers showed improvements following Oltipraz treatment and cell transfer (Figure 7M–P), only semi‐recovery was achieved (compare Figure 3C–F). A partial increase in dPMN‐MDSC levels was consistent with these fetal observations (compare Figure 3H and Figure 7Q). In contrast, no significant differences were observed in dM‐MDSCs, splenic PMN‐MDSCs, splenic M‐MDSCs, or other DICs (Figure S23A–D). Both interventions slightly increased XCR1 expression in dPMN‐MDSCs (compare Figure 3J and Figure 7R) but not in other DICs (Figure S23E). Slight restoration of FOXO1 activity in dPMN‐MDSCs was also observed (Figure 7S–U; Figure S23F), and the partial recovery of nuclear FOXO1 led to a slight increase in downstream target expression and mitochondrial dependency (Figure 7V; Figure S23G,H). In addition, Oltipraz treatment or cell transfer slightly upregulated *Fatp1* and *Fatp2* in dPMN‐MDSCs (Figure S23I), and these treatments did not change XCL1 expression in trophoblasts (Figure S23J,K). Notably, no significant differences were observed between direct Oltipraz treatment and cell transfer (Figure 7M–V; Figure S23A–K). Collectivly, these data demonstrate that Oltipraz promotes fetal growth by restoring the XCR1–FOXO1–OXPHOS axis and rectifying the associated metabolic deficits in decidual XCR1^+^PMN‐MDSCs.

### 2.8 Metabolic Imbalance Mediated by Reduced FOXO1 Activity in Decidual XCR1+PMN‐MDSCs is Associated With FGR in Cases of AMA

**FIGURE 8:**
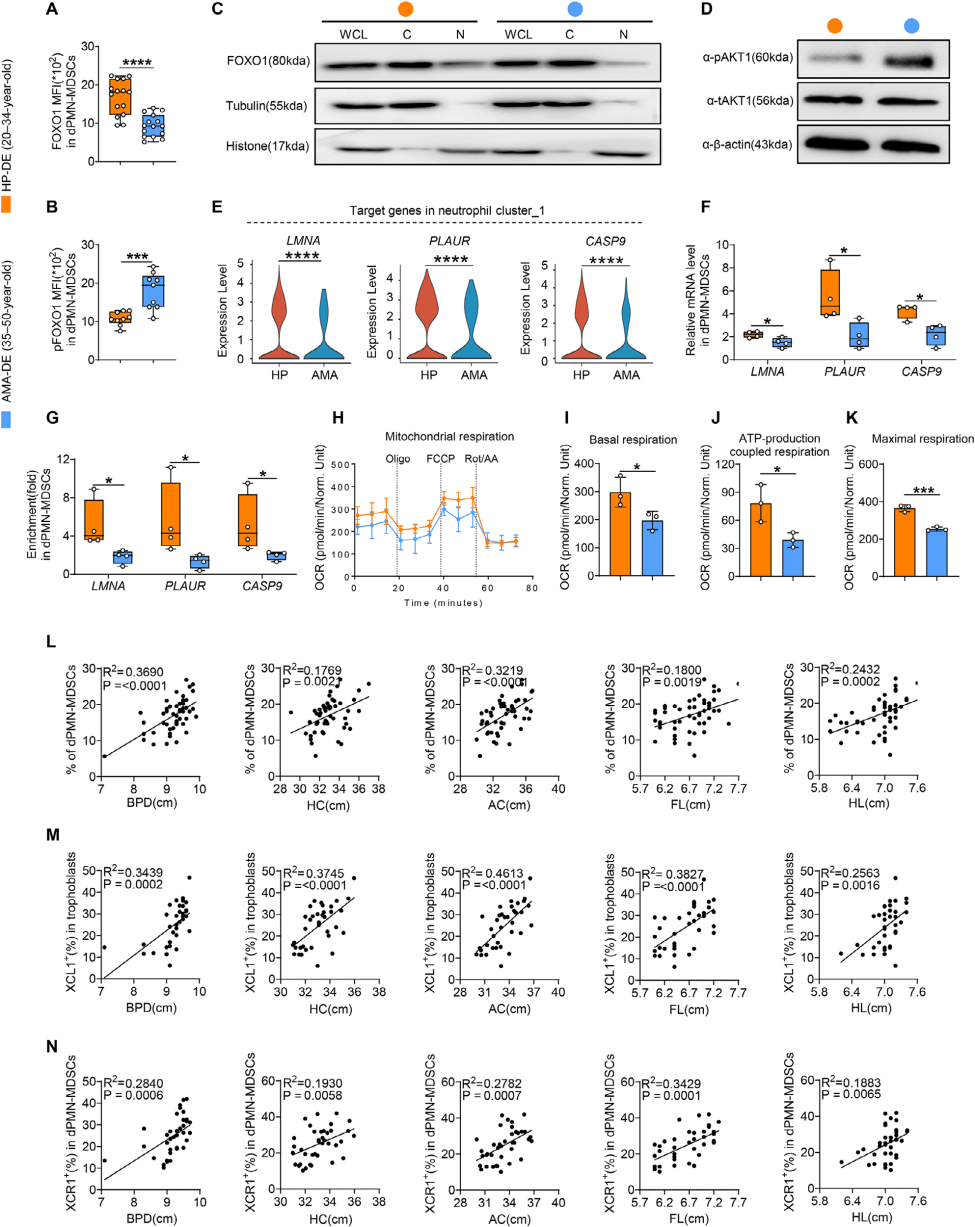
Metabolic imbalance mediated by reduced FOXO1 activity in decidual XCR1^+^PMN‐MDSCs is associated with FGR in cases of AMA. (A, B) MFI of FOXO1 (A, *n =* 15) and pFOXO1 (B, *n =* 9) in human dPMN‐MDSCs. (C) Western blot analysis of whole‐cell lysate (WCL), cytoplasmic (C), and nuclear (N) distributions of FOXO1 expression in decidual XCR1^+^PMN‐MDSCs (replicated twice). (D) Western blot analysis of p‐ and t‐AKT1 in decidual XCR1^+^PMN‐MDSCs (replicated three times). (E) *LMNA*, *PLAUR*, and *CASP9* expression levels in neutrophil cluster_1. (F) mRNA expression levels of the indicated genes in decidual XCR1^+^PMN‐MDSCs (*n =* 4 replicates). (G) Enrichment of FOXO1 to the promoters of *LMNA*, *PLAUR*, and *CASP9* in decidual XCR1^+^PMN‐MDSCs (*n =* 4 replicates). (H–K) Basal OCR, ATP production, and maximal respiratory capacity of decidual XCR1^+^PMN‐MDSCs (*n* = 3 replicates). (L–N) Correlations between dPMN‐MDSC (L, *n =* 51), XCL1^+^trophoblast (M, *n =* 36), decidual XCR1^+^PMN‐MDSC (N, *n =* 38) levels and biparietal diameter (BPD), head circumference (HC), abdominal circumference (AC), femur length (FL), and humerus length (HL). Data are presented as mean ± SEM. Each dot represents a single individual. ns, not significant; ^*^ *p* < 0.05, ^**^ *p* < 0.01, ^***^ *p* < 0.001, ^****^ *p* < 0.0001. Statistical significance was determined using a Student's *t‐*test (A, F, and I–K), Mann–Whitney test (B and G), Wilcoxon test (E), or Pearson's correlation coefficient (L–N).

To assess the clinical relevance of FOXO1‐driven metabolic reprogramming in dPMN‐MDSCs and the correlation between the XCL1–XCR1 axis and fetal growth, we collected decidual tissues from HP and AMA individuals. The results showed significantly reduced FOXO1 expression and increased pFOXO1 levels in dPMN‐MDSCs from the AMA group (Figure 8A,B). Western blot analysis confirmed the altered subcellular localization of FOXO1 in decidual XCR1^+^PMN‐MDSCs from the AMA group, demonstrating decreased nuclear FOXO1 and increased cytoplasmic FOXO1 (Figure 8C), accompanied by an increase in pAKT1 levels (Figure 8D). Analysis of our human scRNA‐seq data revealed significant downregulation of *LMNA*, *PLAUR*, and *CASP9* in the neutrophil cluster_1 (Figure 8E), which was confirmed using qPCR (Figure 8F). After selecting optimal binding sites (Figure S24A,B), ChIP‐qPCR results indicated that FOXO1 binding to the promoter regions of these genes was compromised in decidual XCR1^+^PMN‐MDSCs from the AMA group (Figure 8G). Both SCENITH and Seahorse assays demonstrated weakened mitochondrial activity and enhanced glycolytic capacity in decidual XCR1^+^PMN‐MDSCs from this group (Figure 8H–K; Figure S24C–G). Moreover, clinical regression analyses revealed that biparietal diameter (BPD), head circumference (HC), abdominal circumference (AC), femur length (FL), and humerus length (HL) were significantly positively correlated with dPMN‐MDSC, XCL1^+^trophoblast, and decidual XCR1^+^PMN‐MDSC levels (Figure 8L–N). Collectively, these data indicate that in women of AMA, impaired FOXO1 activity likely contributes to metabolic imbalance in decidual XCR1^+^PMN‐MDSCs. Furthermore, XCL1–XCR1 axis expression is positively correlated with fetal growth.

## 3 Discussion

Communications between trophoblasts and immune cells at the maternal–fetal interface [22, 23, 38], as well as the accumulation of dPMN‐MDSCs in the third trimester [18, 21], are hallmark features of a healthy pregnancy. AMA significantly increases the risk of adverse outcomes, with the maternal immune system being a key link [49]. Here, we report that the interaction between trophoblasts and dPMN‐MDSCs through the XCL1–XCR1 axis is crucial for fetal development during the third trimester. Disruption of the XCL1–XCR1 axis impairs FOXO1 activity and leads to metabolic imbalance in decidual XCR1^+^PMN‐MDSCs, triggering spontaneous FGR in AMA and pregnant *Xcr1* ^–^ *^/^* ^–^ mice. Notably, restoring the expression of the XCL1–XCR1 axis or FOXO1 significantly enhances OXPHOS‐related targets in dPMN‐MDSCs derived from AMA or pregnant *Xcr1* ^–^ *^/^* ^–^ mice. Furthermore, adoptive transfer of decidual XCR1^+^PMN‐MDSCs or perinatal treatment with rXCL1/Oltipraz improves fetal growth by specifically reinstating the XCR1–FOXO1–OXPHOS axis in dPMN‐MDSCs (Figure S25).

In this study, the late‐gestational specificity of XCL1–XCR1–FOXO1‐mediated metabolic reprogramming in dPMN‐MDSCs aligns with stage‐specific changes in dPMN‐MDSC abundance [18]. Specifically, dPMN‐MDSCs are rare early in pregnancy, accumulate during mid‐gestation, and peak in the third trimester, matching the activation kinetics of this pathway. This coordination indicates that the XCL1–XCR1–FOXO1 axis is not constitutively active, but is developmentally regulated and activated when dPMN‐MDSCs reach sufficient numbers to fulfill their essential late‐gestational functions.

The XCL1–XCR1 axis is well established in early pregnancy, in which dNK cell‐derived XCL1 and XCR1‐expressing cDC1s or trophoblasts play critical roles in embryo implantation and trophoblast invasion [16, 50, 51]. Our results reveal a gestational stage‐specific reconfiguration during late gestation: XCR1 expression shifts from cDC1s to selective enrichment in dPMN‐MDSCs, while XCL1 production switches from dNK cells to trophoblasts. This functional rewiring reflects the changing demands of the decidual microenvironment, initially supporting invasive placentation early and later activating dPMN‐MDSCs to sustain fetal growth. This shift also coincides with known alterations in decidual immune composition, characterized by sharp declines in dNK cells and decidual macrophages during mid‐ and late gestation [52, 53, 54]. The minimal XCR1 expression on cDC1s and other DICs during late gestation highlights the specificity of this axis for dPMN‐MDSCs, ensuring the metabolic reprogramming of these cell populations essential for late‐pregnancy homeostasis.

The stage‐specific activation of dPMN‐MDSCs is further evidenced by the secretion of GPFs, such as OPN, OGN, and PTN. These GPFs are vital for fetal development by regulating key processes including tissue growth, collagen synthesis, and skeletal maturation [38, 39, 40, 41]. Thus, the late‐gestational activation of the XCL1–XCR1–FOXO1 axis and the associated GPF secretion by dPMN‐MDSCs represent a coordinated adaptation to meet the demands of late pregnancy and underscore their essential roles in supporting fetal development during this critical period.

While lipid metabolism supports early dPMN‐MDSC differentiation and immune tolerance [35], the signals behind their late‐gestational metabolic shift are still unclear. Here, we identify the XCL1–XCR1 axis as a third‐trimester‐specific pathway that switches dPMN‐MDSCs to OXPHOS, an adaptation to meet the high energy demand of the fetus under hypoxia. This metabolic transition coincides with dPMN‐MDSC accumulation and peak GPF secretion, reflecting coordinated responses to changing gestational needs. Mechanistically, we define a FOXO1‐driven transcriptional network in decidual XCR1^+^PMN‐MDSCs. FOXO1 directly binds to and upregulates Lmna, Plaur, and Casp9, key regulators of mitochondrial function and OXPHOS [55, 56]. Impairment of this pathway in AMA or pregnant *Xcr1* ^–/–^ mice reduces dPMN‐MDSC levels and OXPHOS capacity, linking metabolic dysfunction to FGR. Notably, the Nrf2 activator Oltipraz rescues FGR in AMA mice by selectively activating OXPHOS in decidual XCR1^+^PMN‐MDSCs, without affecting other DICs. Thus, our results confirm both the specificity and therapeutic potential of the XCL1–XCR1–FOXO1 axis.

Our findings extend recent insights into the importance of cellular metabolism for pregnancy success [57, 58, 59]. For instance, impaired autophagy in DSCs disrupts dNK cell function and leads to spontaneous abortion [60], while enhanced glycolysis and OXPHOS in dNK cells promote IFN‐γ and VEGFα secretion, supporting a successful pregnancy [61]. In this study, we identify a distinct FOXO1–OXPHOS regulatory axis in dPMN‐MDSCs that functions specifically in late gestation without affecting early pregnancy. Our study demonstrates that dPMN‐MDSCs are regulated by gestational stage‐specific metabolic signals, offering a new perspective on immune adaptation during pregnancy. Future longitudinal studies are needed to track the dynamic metabolic reprogramming of dPMN‐MDSCs across gestation.

In summary, our study establishes a novel XCL1–XCR1‐mediated crosstalk between trophoblasts and dPMN‐MDSCs. Reduced XCL1‐stimulated decidual XCR1^+^PMN‐MDSCs impair FOXO1 activity, induce metabolic imbalance, and contribute to adverse outcomes associated with AMA. This study lays the groundwork for the development of PMN‐MDSC‐based immunotherapeutic strategies.

## 4 Experimental Section

### 4.1 Human Participants

For clinical analysis (Figure 1A), data from 400 cases of healthy pregnancies and pregnancies with AMA (HP and AMA, respectively; n = 200 cases/group) were collected between January 2024 and November 2025 from Nanfang Hospital of Southern Medical University (Guangzhou, China). Blood and placental samples were obtained from Nanfang Hospital of Southern Medical University (Guangzhou, China) for human experiments. According to current clinical guidelines, HP samples were collected from individuals aged 20–34 years, and AMA samples were collected from pregnant individuals aged 35–50 years. The gestation period was divided into three stages:the first (< 13 weeks), second (14–27 weeks), and third (> 28 weeks) trimesters. FGR refers to an estimate of fetal weight, head and abdomen circumferences, and femur and humerus lengths below the 10th percentile for gestational age. Human scRNA‐seq samples were obtained from a single individual, for whom clinical characteristics are listed in Table S1.

The exclusion criteria included ectopic pregnancy, PE, chronic hypertension, chronic hypertension complicating pregnancy, diabetes, GDM, pregnancies with FGR in women aged 20–34 years, anemia, obesity (BMI ≥ 30 kg/m^2^), acute infectious diseases, cardiovascular diseases, severe inflammation, subclinical inflammation, chronic diseases, and autoimmune diseases. The clinical characteristics of the HP and AMA cohorts are presented in Table S2. For clinical indicators with multiple test results during pregnancy, the last available result prior to delivery was collected. For the clinical regression analyses, multivariate linear regression models adjusted for potential confounding variables were used. The corresponding analyses are provided in Tables S3–S5. To validate the robustness of the models, multicollinearity was evaluated using the variance inflation factor, and the model assumptions, including linearity, independence, normality, and homoscedasticity, were systematically tested. These diagnostic analyses are summarized in Tables S6–S9. The model assumptions were further visualized using the following plots: residual vs. fitted value (scatter plot), normal Q–Q plot of residuals, distribution of residuals, and standardized residual vs. fitted values, which are presented in Figure S26.

A more detailed clinical profile, including maternal metrics, inflammation biomarkers, metabolic panels (e.g., glucose tolerance tests, blood lipid profiles, blood pressure, and liver function tests), and standard blood/urine tests is provided in Supporting Table. This study was approved by the Clinical Ethics Review Committee of Nanfang Hospital of Southern Medical University. Written informed consent was obtained from all participants or their legal guardians after admission (approval number: NFEC‐2024‐503).

### 4.2 Human Sample Processing and Isolation

For primary whole blood cell isolation, blood was diluted with phosphate‐buffered saline (PBS), supplemented with red blood cell (RBC) lysis buffer (TONBO), and washed with PBS. The cells were then isolated for further use.

Primary decidual mononuclear cells were isolated as previously described [18, 21]. Fresh placental samples were macroscopically dissected from the central region of the maternal‐facing surface to separate the decidual layers. The layers were cut into small pieces and digested with 1 mg/mL collagenase type IV (Gibco) and 200 U/mL DNase I (Solarbio) in Roswell Park Memorial Institute (RPMI)‐1640 medium (Basal Media) for 1.5–2 h at 37°C. Thereafter, RBCs were removed from the suspensions and filtered through a 70 µm cell filter. Decidual mononuclear cells were then isolated using 40/80% Percoll density gradient centrifugation (Cytiva). The isolated cells were washed with PBS for further use.

Primary trophoblasts were isolated as previously described, with minor modifications [53, 62]. The villous tissues were separated from the basal membrane, cut into small pieces, added to Dulbecco's modified eagle's medium (DMEM)/Ham's F12 medium (Basal Media) containing 10% fetal bovine serum (FBS), 0.25% Trypsin‐EDTA solution (Gibco), and 1 mg/mL collagenase type IV (Gibco), and digested at 37°C for 20 min. After removing the RBCs, the cells were layered on a Ficoll gradient (Serumwerk Bernburg) for density gradient centrifugation. The isolated cells were then incubated in a tissue culture dish at 37°C for 30 min to remove the adherent macrophages. The suspension was prepared for subsequent tests.

### 4.3 Mouse Strains

8–12‐week‐old wild‐type males and females and 24–32‐week‐old wild‐type females with a C57BL/6 background were purchased from the Center of Laboratory Animals of Southern Medical University. *Xcr1^−/−^* females were created by crossing *Xcr1^−/−^* males with *Xcr1* ^+/−^ females (Shanghai Model Organisms Center, Inc). *Foxo1* ^fl/fl^ mice were supplied by Professor Zhexiong Lian from Guangdong Provincial People's Hospital (Guangdong Academy of Medical Sciences), and *S100a8* ^cre^ mice were gifted by Cyagen Biosciences, Inc. *Foxo1* ^fl/fl^ *S100a8* ^cre^ females were generated by crossing *Foxo1* ^fl/fl^ *S100a8* ^cre^ males with *Foxo1* ^fl/fl^ females.

### 4.4 Mouse Models and Detailed Treatments

For recombinant XCL1 (rXCL1) treatment, 24–32‐week‐old AMA or 8–12‐week‐old pregnant *Xcr1^−/−^* mice were injected (i.p.) with 0.2 µg rXCL1 (R&D systems) or PBS per day from E13.5 to E16.5 [63]. For Oltipraz treatment, 24–32‐week‐old AMA or 8–12‐week‐old pregnant *Xcr1^−/−^* mice were injected (i.p.) with 2.0 mg/kg Oltipraz (Selleck) dissolved in 100 µL of dimethyl sulfoxide (DMSO) or an equivalent volume of DMSO per day from E13.5 to E16.5 [44, 45].

The day a copulation plug was observed was designated E0.5; mice were weighed, recorded from E0.5 to E18.5, and euthanized on E18.5. Fetal indicators included fetal weights, crown‐to‐rump, and tail lengths. All mice had free access to tap water and standard rodent particle food and were housed in a specific pathogen‐free environment (humidity, 55 ± 5%; temperature, 23 ± 2°C; 12 h:12 h dark/light cycle). All experimental animal procedures adhered to the Animal Experiments Ethics Requirements of Southern Medical University, and approval for the animal studies was obtained from the Southern Medical University Experimental Animal Ethics Committee (approval number: L2023083).

### 4.5 Mouse Sample Processing and Isolation

For primary decidual mononuclear cell isolation [18, 21], the decidual layers were separated carefully from the placenta and digested in DMEM (Basal Media) with 100 U/mL hyaluronidase (Sigma), 0.5 mg/mL collagenase type IV (Gibco), and 200 U/mL DNase I (Sangon Biotech) at 37°C for 30 min. The single‐cell suspension was lysed in ACK lysis buffer and filtered through a 70 µm cell filter. Decidual mononuclear cells were then isolated using 30/70% Percoll density gradient centrifugation (Cytiva) and prepared for subsequent experiments. For primary trophoblast isolation [53, 62], the trophoblast layers were carefully separated from the decidua basalis. The obtained tissues were added to DMEM/Ham's F12 medium (Basal Media) containing 10% FBS, 0.25% Trypsin‐EDTA solution (Gibco), and 0.5 mg/mL collagenase type IV (Gibco) and digested at 37°C for 15 min. After removing the RBCs, the cells were incubated in a tissue culture dish at 37°C for 30 min. The suspension was collected and prepared for subsequent tests.

### 4.6 Flow Cytometry

For surface staining, prepared cells (1 × 10^6^) were pre‐incubated with 0.5 µg purified CD16/32 antibody to block Fc‐mediated interactions. Cells were then stained with a cocktail containing Ghost Dye Violet 510 (TONBO) and cell surface antibodies diluted in 50 µL FACS buffer at 4°C for 30 min. For intracellular staining, the cells were stained with surface markers, fixed and permeated with Foxp3/transcription factor staining buffer (eBioscience) at 4°C for 30 min, and stained with target antibodies. For intracellular GPF staining, the cells were pre‐stimulated with 50 ng/mL phorbol‐12‐myristate‐13‐acetate (Sigma–Aldrich), 1 µg/mL ionomycin (Sigma–Aldrich), and 1 µg/mL brefeldin A (Sigma–Aldrich) in RPMI‐1640 medium (Basal Media) containing 10% FBS at 37°C for 4 h and processed as described above. The antibodies used in this study are detailed in Table S10.

The gating strategies for human samples are detailed in Figure S27. The classifications are as follows: LOX1^+^PMN‐MDSCs (Live CD45^+^CD14^−^CD15^+^LOX1^+^), XCR1^+^PMN‐MDSCs (Live CD45^+^CD14^−^CD15^+^LOX1^+^XCR1^+^), cDC1s (Live CD45^+^CD1c^−^ CD3^−^CD14^−^CD19^−^CD20^−^CD56^−^CD123^−^CD11c^+^CD141^+^MHCII^+^IRF8^+^Clec9A^+^), cDC2s (Live CD45^+^CD3^−^CD14^−^CD19^−^CD20^−^CD56^−^CD123^−^MHCII^+^CD11c^+^CD1c^+^), cDCs (Live CD45^+^CD3^−^CD19^−^CD14^−^MHCII^+^CD11c^+^CD206^+^Zbtb46^+^), dNK cells (Live CD45^+^CD3^−^ CD19^−^CD56^+^CD49a^+^), Th cells (Live CD45^+^CD3^+^CD4^+^CD8^−^), CTL (Live CD45^+^CD3^+^ CD8^+^CD4^−^), dMφ (Live CD45^+^CD14^+^HLA‐DR^+^CD68^+^), type 1 innate lymphoid cells (ILC1s, Live CD45^+^CD3^−^CD8^−^CD14^−^CD19^−^CD34^−^CD56^−^CD123^−^CD11b^−^Ly6G^−^CD11c^−^FceR1α^−^ TCRαβ^−^B220^−^TCRγδ^−^CD127^+^CD161^+^CRTH2^+^CD117^+^), DSCs (Live CD45^−^Vimentin^+^), and trophoblasts (Live CD45^−^HLA‐G^+^).

The gating strategies for murine samples are detailed in Figure S28. The classifications are as follows: PMN‐MDSCs (Live CD45^+^CD11b^+^Ly6G^+^Ly6C^−/low^), XCR1^+^PMN‐MDSCs (Live CD45^+^CD11b^+^Ly6G^+^Ly6C^−/low^XCR1^+^), cDC1s (Live CD45^+^CD3^−^ CD9^−^B220^−^Ly6G^−^NK1.1^−^Ter119^−^CD19^−^F4/80^−^CD11b^−^MHCII^+^CD11c^+^CD103^+^IRF8^+^Clec9A^+^), cDC2s (Live CD45^+^CD3^−^CD14^−^CD19^−^NK1.1^−^CD20^−^CD123^−^CD103^−^MHCII^+^CD11c^+^ CD11b^+^), cDCs (Live CD45^+^CD3^−^CD19^−^SiglecF^−^MHCII^+^CD11c^+^Zbtb46^+^), dNK cells (Live CD45^+^CD3^−^CD19^−^NK1.1^+^CD49a^+^), Th cells (Live CD45^+^CD3^+^CD4^+^CD8^−^), CTL (Live CD45^+^CD3^+^CD8^+^ CD4^−^), dMφ (Live CD45^+^CD11b^+^F4/80^+^), ILC1s (Live CD45^+^CD3^−^CD4^−^ CD5^−^CD8^−^B220^−^CD11b^−^Ly6G^−^TER119^−^CD11c^−^NK1.1^−^TCRβ^−^TCRγδ^−^T‐bet^+^CD127^+^), DSCs (Live CD45^−^Vimentin^+^), and trophoblasts (Live CD45^−^CD31^+^cytokeratin‐7^+^).

### 4.7 Cell Purification

Human PMN‐MDSCs were labeled with LOX1‐PE (BioLegend) or XCR1‐PE (BioLegend) and sorted using an EasySep Human PE Positive Selection Kit II (StemCell). Murine PMN‐MDSCs were labeled with Ly6G‐PE (eBioscience) or XCR1‐PE (BioLegend) and purified using the EasySep Mouse PE Positive Selection Kit II (StemCell). Human trophoblasts were labeled with HLA‐G‐PE (eBioscience) and subjected to the EasySep Human PE Positive Selection Kit II. Murine trophoblasts were labeled with cytokeratin 7‐unconjugated monoclonal antibody (eBioscience) and goat anti‐mouse IgG‐PE secondary antibody (eBioscience) followed by an EasySep Mouse PE Positive Selection Kit II (StemCell). The purity of the sorted cells was evaluated by flow cytometry (Figure S29), showing that at least 70% of the cells were positive for target markers.

### 4.8 Adoptive Transfer

The following cells were purified as described above: (1) Splenic neutrophils from wild‐type non‐pregnant mice, (2) dPMN‐MDSCs from pregnant *Xcr1* ^+/+^ or *Xcr1^−/−^* mice, and (3) dPMN‐MDSCs from Oltipraz‐treated AMA or pregnant *Xcr1^−/−^* mice. The sorted cells (2 × 10^5^/mouse) were suspended in 150 µL of PBS and injected (i.v.) into the recipient mice on E13.5 [18, 21]. At E18.5, all recipient mice were euthanized and analyzed.

### 4.9 Trophoblast Culture and Supernatant Assay

Primary human or murine trophoblasts were re‐suspended in DMEM/Ham's F12 medium (Basal Media) containing 10% FBS and cultured in a 96‐well plate coated with fibronectin for 6 h [53, 62]. The supernatant was collected for further use or determined using an ELISA kit (Dogesce) according to the manufacture's protocol.

### 4.10 dPMN‐MDSC Culture and Supernatant Assay

Primary human or murine dPMN‐MDSCs were cultured (1) alone; (2) with trophoblast‐cultured supernatant; (3) with 50 ng/mL phorbol‐12‐myristate‐13‐acetate (Sigma–Aldrich), 1 µg/mL ionomycin (Sigma–Aldrich), 1 µg/mL brefeldin A (Sigma–Aldrich), and 500 ng/mL rXCL1; and (4) with trophoblast‐cultured supernatant and 1000 ng/mL XCR1 mAb (PeproTech) at 37°C for 6 h. The supernatant was collected and determined using an ELISA kit (Dogesce) according to the manufacturer's protocol.

### 4.11 Single‐Cell Metabolism Analysis of Translation Inhibition (SCENITH)

Decidual mononuclear single‐cell suspensions obtained from the decidua (density: 1 × 10^6^ cells/mL) were treated with DMSO, 50 mM 2‐deoxy‐D‐glucose (Selleck), 2 µM oligomycin (Selleck), or a combination of both drugs at 37°C for 40 min and subsequently treated with 10 µg/mL puromycin (Selleck) at 37°C for 30 min. The cells were collected, incubated with surface markers, and fixed and permeabilized. Intracellular staining was performed using an anti‐puromycin antibody (Sigma–Aldrich) at 4°C for 30 min and resuspended in FACS buffer for flow cytometric analysis [64].

### 4.12 Seahorse Assay

dPMN‐MDSCs at 1 × 10^4^ cells per well were seeded into poly‐D‐lysine (Beyotime)‐coated XF96 cell‐culture microplates. The cells were pre‐incubated at 37 °C for 1 h in a non‐CO_2_ incubator in an assay medium (pH 7.4) with 1 mM sodium pyruvate, 2 mM glutamine, and 10 mM glucose. The OCR and ECAR were measured using the Seahorse XFe96 Analyzer (Agilent Technologies). Three consecutive measurements of the OCR (pmol min^−1^) were obtained under basal conditions and in response to 2 µM oligomycin (Oligo), 1.5 µM carbonyl cyanide‐p‐trifluoromethoxy phenylhydrazone (FCCP), and 1 µM antimycin A/rotenone (Rot/AA). The basal and maximal OCR were calculated before Oligo addition and after FCCP injection, respectively. ATP‐linked respiration was calculated by subtracting the OCR after Oligo addition from the basal OCR. Three consecutive measurements of the ECAR (mpH min^−1^) were obtained under basal conditions and in response to 1 µM Rot/AA and 20 mM 2‐deoxy‐D‐glucose (2‐DG). Basal glycolysis was measured under basal conditions. Compensatory glycolysis was determined after Rot/AA supplementation [34, 44]. Data were analyzed using Wave software (Agilent v2.6.1).

### 4.13 T‐Cell Proliferation Assay

In the human assay, prepared primary whole blood cells were considered a co‐cultured group (TC), including T cells and PMN‐MDSCs. TC^depletion^ cells (excluding PMN‐MDSCs) were obtained from the first wash supernatant during PMN‐MDSC sorting. The TC^depletion^ and TC groups were stimulated with anti‐CD3/CD28 functional antibodies (TONBO), and unstimulated TC^depletion^ was used as the negative control.

In the mouse assay, splenic T cells were labeled with CD3‐PE (eBioscience), purified using the EasySep Mouse PE Positive Selection Kit II (StemCell), and stained with carboxyfluorescein succinimidyl amino ester (Invitrogen) at 37°C for 15 min. Sorted primary T cells were incubated with PMN‐MDSCs at different ratios (T cells:PMN‐MDSCs = 1:0, 2:1, 4:1, and 8:1) under 5 mg/mL concanavalin A (Sigma) stimulation at 37°C for 3 days; unstimulated T cells served as the negative control. The cells were then resuspended in FACS buffer for flow cytometric analysis.

### 4.14 Hematoxylin–Eosin Staining

Human and murine placental tissues were fixed with 4% paraformaldehyde overnight, followed by dehydration, paraffin embedding, and sectioning at a thickness of 4 µm along the sagittal plane. The sections were stained with hematoxylin and eosin (Servicebio) and imaged using a microscope (Nikon). The inflammatory cell infiltration was determined blindly by two independent observers. In the murine samples, a scoring system from zero to three was applied based on inflammatory cell counts per high‐power field (HPF). The three most inflamed fields in the decidua were selected at 400 × magnification, and the number of inflammatory cells per field was counted. A score of zero was assigned for < 5, one for 5–10, two for 10–49, and three for 50 or more cells [65]. In the human samples, grade zero was assigned for < 49 inflammatory cells per HPF, grade one for ≥ 50 inflammatory cells per HPF, grade two for the presence of diffuse inflammatory cells in one tissue section, and grade three for confluent or widespread inflammation [66].

### 4.15 RNA Isolation and Quantitative Real‐Time PCR (qRT‐PCR)

Total RNA was extracted using TRIzol reagent (ECOTOP) and cDNA was generated using the StarScript II First‐strand cDNA Synthesis kit (GenStar). For qRT‐PCR, cDNA amplification was conducted using RealStar Green Power Mixture (Genstar) and the QuantStudio 6 Flex system (Applied Biosystems). The primer sequences used in this study are listed in Table S11.

### 4.16 ChIP‐qPCR Assay

ChIP‐qPCR assay was performed as previously described [21]. Sorted dPMN‐MDSCs were crosslinked with 1% paraformaldehyde and quenched using 125 mM glycine. After sonicating the sheared chromatin, input samples were collected from the supernatant. Immunoprecipitation was performed using an anti‐FOXO1 antibody (Cell Signaling Technology) or a normal rabbit IgG (Cell Signaling Technology) at 4°C overnight. After adding Pierce Dynabeads Protein A/G (Invitrogen), the sample was eluted and de‐crosslinked, and DNA was extracted and purified for further qPCR analysis. The primer sequences are listed in Table S12.

### 4.17 Dual‐luciferase Reporter Assay

HEK‐293T cells were seeded at a density of 5 × 10^4^ cells/well and co‐transfected as follows: (1) pCDNA3.1‐3×HA‐EV and pFR‐luc (*Lmna*, *Plaur*, and *Casp9*), (2) pCDNA3.1‐3×HA‐FOXO1 and pFR‐luc, (3) pCDNA3.1‐3×HA‐FOXO1, pFR‐luc, and pCDNA3.1‐Flag‐XCL1, (4) pCDNA3.1‐3×HA‐FOXO1, pFR‐luc, and pCDNA3.1‐Flag‐XCR1, and (5) pCDNA3.1‐3×HA‐FOXO1, pFR‐luc, pCDNA3.1‐Flag‐XCL1, and pCDNA3.1‐Flag‐XCR1. For the FOXO1 knockdown experiment, HEK‐293T cells were co‐transfected as follows: (1) pCDNA3.1‐3×HA‐EV and pFR‐luc, (2) pCDNA3.1‐3×HA‐FOXO1 and pFR‐luc, (3) si‐FOXO1 and pFR‐luc, and (4) pCDNA3.1‐3×HA‐FOXO1, si‐FOXO1, and pFR‐luc. The *Renilla* luciferase vector was used to monitor transfection efficiency. After 36 h of transfection, the cells were lysed and luciferase activity was measured using a Dual Luciferase Reporter Assay Kit (Vazyme) according to the manufacturer's protocol. The luciferase activity was quantified in relative light units. The average firefly luciferase (*Photinus pyralis*) activity was normalized to that of the *Renilla* luciferase [67]. The sequences are listed in Table S13.

### 4.18 AdV Transfection Assay

Primary dPMN‐MDSCs and trophoblasts isolated from AMA mice were seeded at a density of 5 × 10^4^ cells/well. After 6 h of incubation at 37°C, trophoblasts were treated with AdV‐XCL1 and cultured for 24 h. Subsequently, the culture supernatant and cells were collected for further use. For dPMN‐MDSC transfection, specific AdV or AdV‐XCL1‐conditioned supernatant was added. After an 800‐rpm centrifugation for 30 min at 37°C, the cells were cultured at 37°C for 36 h and collected for further analysis. All AdV were used at a multiplicity of infection of 200. After transfection, the cells viability was at least 80%. The transfection efficiency was detected using a microscope (Nikon) and calculated as the ratio of fluorescent‐positive cells to the total cell count [68].

### 4.19 Nuclear and Cytoplasmic Protein Extraction and Western Blotting Analysis

Nuclear and cytoplasmic proteins were extracted according to the guidelines of the nuclear and cytoplasmic protein extraction kit (Beyotime), and whole cell proteins were lysed in RIPA buffer (Beyotime) supplemented with proteinase and phosphatase inhibitors. Protein concentrations were measured using Bradford assay. Proteins were separated using SDS‐PAGE, transferred to polyvinylidene difluoride membranes (Millipore), blocked with 5% non‐fat dry milk, and incubated with primary antibodies at 4°C overnight. The blot was then labeled with horseradish peroxidase‐conjugated secondary antibodies, detected with a chemiluminescence kit (Millipore), and quantified using Image Lab software. The primary antibodies are listed in Table S10.

### 4.20 Measurements of ATP and Lactic Acid

Intracellular ATP and D‐lactate acid concentrations of freshly sorted dPMN‐MDSCs were measured using ATP assay kits (Solarbio) and D‐lactate colorimetric assay kits (Elabscience), according to the manufacturer's instructions.

### 4.21 RNA‐Sequencing Analysis

RNA‐seq was performed at the Beijing Genomics Institution (Beijing, China). Differential expression analyses were performed using the R package DESeq2 (version 1.44.0), and a *p* < 0.05 cutoff was applied to determine the DEGs. KEGG analysis of DEGs was performed using the R package clusterProfiler (version 4.12.6).

### 4.22 ScRNA‐seq Analysis

Human scRNA‐seq data were obtained from NovelBio Co., Ltd. using the CytoNavigator SingleCell Analysis Platform (sc.novelbrain.com). Murine scRNA‐seq data were obtained from the National Center for Biotechnology Information Sequence Read Archive under the accession number PRJNA1048538 (https://www.ncbi.nlm.nih.gov/bioproject/PRJNA1048538) [42]. FASTQ was used to remove adapter contamination and filter low‐quality reads to obtain clean data. Single‐cell transcriptome analysis was performed using UMI‐tools to identify the cell barcode whitelists. Using the STAR map of custom parameters in the UMI‐tools standard pipeline, UMI‐based clean data were mapped to the human genome (Ensemble version 104) or the murine genome (Ensemble version GRCm39) to obtain UMI counts for each sample. Downstream analyses were performed using the Seurat package (version for human data: 4.1.1; version for murine data: 5.2.1, https://satijalab.org/seurat/), including DEGs (Log2FC > 0.25; *p*‐value < 0.05), violin plots, and bubble plots for the genes of interest. Pseudotime trajectory analysis was performed using the Monocle 3 package (version 1.4.26). For gene enrichment analysis, Fisher's exact test was used to calculate the *p*‐value for each gene set. Raw *p*‐values were adjusted for multiple hypothesis tests using the Benjamini–Hochberg method. Enrichment analysis was applied to the KEGG annotation (201900613). Significant differences in gene expression were analyzed using the ggplot2 package (version 0.6.4).

### 4.23 CUT&Tag Processing and Sequencing

For sample preparation of CUT&Tag, nuclei were isolated according to a published protocol using a minimum of 10^5^ nuclei. Nuclei were resuspended in 200 µL buffer containing 1 µg FOXO1 antibody or IgG and incubated at 4°C overnight. The Hyperactive Universal CUT&Tag Assay Kit for Illumina Pro was used to perform CUT&Tag analysis. Qubit Flex was used for initial quantitation after the library was constructed, and the library size range was checked using an Agilent 4200 Tapestation to ensure the inserted fragment size. After qualified library inspection, different libraries were pooled according to effective concentration and target data volume requirements, and the Novaseq 6000 PE150 mode was used for sequencing.

### 4.24 Statistical Analysis

Data are presented as mean ± SEM. Statistical significance is defined as *p* < 0.05, and “ns” indicates non‐significant results in figures. Detailed statistical methods for each experiment are provided in the figure legends. Routine analyses—including two‐group and multiple‐group comparisons and Pearson's correlation—were performed using GraphPad Prism (v10.1.2). Multivariable linear regression, model assumption evaluation, and data visualization were conducted in R using the broom package (v1.0.10) and ggplot2 (v4.0.0), respectively. Prior to statistical analysis, normality and homoscedasticity were assessed using the Shapiro‐Wilk test and Levene's test, respectively; the results determined the use of parametric or non‐parametric methods. For two‐group comparisons, an unpaired Student's *t*‐test was used when assumptions were met; otherwise, the Mann–Whitney (unpaired) or Wilcoxon signed‐rank (paired) tests were applied. For multiple‐group comparisons, a one‐way ANOVA followed by Tukey's test (equal variances) or Dunnett's T3 test (unequal variances) was used for parametric data, and the Kruskal‐Wallis test followed by Dunn's post hoc test was used for non‐parametric data. Clinical correlation between two parameters was assessed using Pearson's correlation coefficient. For multivariable linear regression, an initial model including all candidate independent variables was built. Variables with a variance inflation factor > 10, indicating severe multicollinearity, were excluded. The final refitted model was examined for the statistical assumptions of linearity, residual independence, normality, and homoscedasticity. For each independent variable in the final model, the standardized regression coefficient and its 95% confidence interval were reported; *p* < 0.05 was considered statistically significant. The sign and magnitude of each coefficient indicated the direction and strength of the independent association, respectively.

## Author Contributions

M.C., Y.G., Q.Z., and J.L. contribute equally to this work. Y.H. and M.C. designed the research. M.C., Y.G., Q.Z., and J.L. performed the experiments and analyzed the data. S.K., Q.Z., and Z.H. provided technical assistance in the clinical samples and animal experiments. C.L., S.X., Z.Z., A.Y., and J.L. acquired the clinical data. M.C., Y.G., and J.L. wrote the manuscript. Y.H., K.W., and J.L. reviewed and edited the manuscript. Y.H., K.W., and J.L. supervised the study. All authors read and approved the manuscript.

## Funding

This study was supported by the following grants: the National Natural Science Foundation of China (grant numbers 82171706 and 82471736), the Guangdong Basic and Applied Basic Research Foundation (grant numbers 2022A1515140172, 2024A1515140189, and 2024A1515012897), and the Open Fund Project of Guangdong Academy of Medical Sciences (grant number YKY‐KF202209).

## Conflicts of Interest

The authors declare that they have no conflicts of interest.

## Supporting information




**Supporting File 1**: advs73746‐sup‐0001‐SuppMat.docx.

**Supporting File 2**: advs73746‐sup‐0002‐SupportingTable.xlsx.

**Supporting File 3**: advs73746‐sup‐0003‐Data.xlsx.

## Data Availability

The data that support the findings of this study are openly available in [Gene Expression Omnibus] at [https://www.ncbi.nlm.nih.gov/geo/query/acc.cgi?acc = GSE288396, https://www.ncbi.nlm.nih.gov/geo/query/acc.cgi?acc = GSE289975, https://www.ncbi.nlm.nih.gov/geo/query/acc.cgi?acc = GSE288405], reference number [288396].
