# Supplementary material for: Metabolic Reprogramming Driven by Trophoblasts and Decidual XCR1+PMN‐MDSC Crosstalk Controls Adverse Outcomes Associated With Advanced Maternal Age
Source: Adv Sci (Weinh). 2026 Jan 12;13(16):e13370. doi: 10.1002/advs.202513370 (PMC13042359; doi:10.1002/advs.202513370)
Supplement: Supplementary file 1 — Supporting File 1: advs73746‐sup‐0001‐SuppMat.docx. [file ADVS-13-e13370-s001.docx]

Supporting Information

Metabolic Reprogramming Driven by Trophoblasts and Decidual XCR1^+^PMN-MDSC Crosstalk Controls Adverse Outcomes Associated with Advanced Maternal Age

Meiqi Chen, Yuxiong Guo, Qing Zhao, Jingping Liu, Shuyi Kuang, Zhengcong Huang, Chenlin Lv, Shuxiu Xu, Zekai Zhuang, Anyan Yang, Jing Li*, Kai Wu*, and Yumei He*

**Figure S1.** Activation of PMN-MDSCs in peripheral blood (PB) in AMA is compromised. A) Percentages of LOX1^+^PMN-MDSCs among CD45^+^ cells in PB during pregnancy (*n* = 30, 40, and 20, respectively). B, C) Proliferation rates of CD4^+^ and CD8^+^ T cells (*n* = 4). D) mRNA expression levels of *S100A8*, *S100A9*, *CYBB*, and *ARG1* in the PMN-MDSCs from PB (*n* = 4 replicates). E) Mean fluorescence intensity (MFI) of S100A9 in the PMN-MDSCs from PB (*n* = 6). F) Western blot analysis for S100A9, phosphorylated-STAT6 (p-STAT6), and total-STAT6 (t-STAT6) in the PMN-MDSCs from PB (replicated three times). Data are presented as mean ± SEM. Each dot represents a single individual. ns, not significant; **p* < 0.05, ***p* < 0.01, ****p* < 0.001, *****p* < 0.0001. Statistical significance was determined using a Student’s *t*-test (A−E) or Mann−Whitney test (A and D).

**Figure S2.** Decidual PMN-MDSCs (dPMN-MDSCs) levels negatively correlate with maternal age; however, no significant changes are observed in dPMN-MDSC levels, fetal development indicators, or inflammation in the circulation and placental tissues of AMA patients. A) Forest plot showing the correlation between dPMN-MDSC levels and multiple dependent variables (HP, *n* = 23. AMA, *n* = 28). B–G) Comparison of dPMN-MDSC levels (B), biparietal diameter (BPD, C), head circumference (HC, D), abdominal circumference (AC, E), femur length (FL, F), and humerus length (HL, G) between male and female infants born to AMA mothers (*n* = 14). H) Secretion levels of IFN-γ, TNF-α, and IL-10 in the plasma of HP and AMA (*n* = 4). I, J) Representative Hematoxylin and Eosin (H&E) staining and statistical analysis of inflammatory cell infiltration among the decidua (*n* = 10, scalar bar: 50 μm). Data are presented as mean ± SEM. Each dot represents a single individual. ns, not significant; **p* < 0.05, ***p* < 0.01, ****p* < 0.001, *****p* < 0.0001. Statistical significance was determined using a Student’s *t*-test (B−H) or Mann−Whitney test (J). D: decidua.

**Figure S3.** Decidual PMN-MDSCs (dPMN-MDSCs) exhibit a distinct gene expression profile. A, B) Heatmap (A) and Kyoto Encyclopedia of Genes and Genomes (KEGG) analysis (B) showing differentially expressed genes (DEGs) in the PMN-MDSCs from PB versus DE (*n* = 3). C, D) mRNA expression levels of chemokine receptor-related (C) and functional (D) genes in PMN-MDSCs (*n* = 4 replicates). Data are presented as mean ± SEM. Each dot represents a single individual. ns, not significant; **p* < 0.05, ***p* < 0.01, ****p* < 0.001, *****p* < 0.0001. Statistical significance was determined using a Student’s *t*-test (D) or Mann−Whitney test (C and D). PB: peripheral blood; DE: decidua; OPN: osteopontin; OGN: osteoglycin; PTN: pleiotrophin.

**Figure S4.** No significant difference in *CCR1* expression in neutrophil clusters_1 between HP and AMA. A) Bubble diagrams depicting the expression of signature genes. B) Uniform Manifold Approximation and Projection (UMAP) visualizing nine major cell types in sorted LOX1^+^ immune cells among the decidua. C) Violin plots showing the expression of marker genes across five clusters of neutrophils. D) Violin plot showing *CCR1* expression in neutrophil cluster_1. E) mRNA expression levels of *CCR1* in dPMN-MDSCs (*n* = 4 replicates). Data are presented as mean ± SEM. Each dot represents a single individual. ns, not significant; **p* < 0.05, ***p* < 0.01, ****p* < 0.001, *****p* < 0.0001. Statistical significance was determined using a Student’s *t-*test (E) or Wilcoxon test (D).

**Figure S5**. XCR1 is specifically expressed in dPMN-MDSCs. A) UAMP visualization of the neutrophil clusters embedded in the pseudotime trajectory using Monocle 3, with cells ordered based on highly dispersed ordering genes. Distinct clusters are color-coded. B) Violin plots showing the expression of neutrophil and canonical DC markers across five identified neutrophil clusters. C, D) Representative flow cytometry plots (C) and percentages (D) of XCR1^+^ cells among various populations (*n* = 8). E) Percentages of XCR1^+^ cells in PMN-MDSCs from PB, decidual immune cells (DICs), and trophoblasts between HP and AMA (*n* = 6). F, G) Violin plots showing the expression of *XCL1* and *XCL2* in neutrophil cluster_1. H) mRNA expression levels of *XCL1* and *XCL2* in dPMN-MDSCs (*n* = 4 replicates). Data are presented as mean ± SEM. Each dot represents a single individual. ns, not significant; **p* < 0.05, ***p* < 0.01, ****p* < 0.001, *****p* < 0.0001. Statistical significance was determined using a Student’s *t-*test (D, E, and H) or Wilcoxon test (F and G).

**Figure S6**. XCL1 is specifically expressed in trophoblasts and shows a reduction in AMA. A) Heatmap illustrating DEGs in trophoblasts from HP and AMA (*n* = 3). B) mRNA expression level of *XCL2* in trophoblasts (*n* = 4 replicates). C) Percentages of XCL1^+^ cells in trophoblasts, DICs, and decidual stromal cells (DSCs) (*n* = 6). Data are presented as mean ± SEM. Each dot represents a single individual. ns, not significant; **p* < 0.05, ***p* < 0.01, ****p* < 0.001, *****p* < 0.0001. Statistical significance was determined using a Student’s *t-*test (B and C).

**Figure S7**. The expression levels of OPN, OGN, and PTN are diminished in dPMN-MDSCs of AMA. A−F) Violin plots showing the expression of OPN (encoded by *SPP1*), *OGN*, *PTN*, vascular endothelial growth factor (*VEGF*), insulin-like growth factor (*IGF2*), and integrin alpha D (*ITGAD*) in neutrophil cluster_1. G) mRNA expression levels of the indicated genes in dPMN-MDSCs (*n* = 4 replicates). H) MFI of OPN, OGN, and PTN in dPMN-MDSCs (*n* = 10). I−K) Secretion levels of OPN, OGN, and PTN in each group (*n* = 4). Data are presented as mean ± SEM. Each dot represents a single individual. ns, not significant; **p* < 0.05, ***p* < 0.01, ****p* < 0.001, *****p* < 0.0001. Statistical significance was determined using a Student’s *t-*test (G−K), Mann−Whitney test (H), or Wilcoxon test (A−F).

**Figure S8**. The activation of splenic PMN-MDSCs in AMA mice is compromised. A–C) Representative flow cytometry plots (A) and percentages of splenic PMN-MDSCs (B) and M-MDSCs (C) in CD45^+^ cells across the groups (*n* = 10). D, E) Proliferation rates of CD4^+^ and CD8^+^ T cells (*n* = 4). F) mRNA expression levels of *S100a8*, *S100a9*, *Cybb*, and *Arg1* in splenic PMN-MDSCs (*n =* 4 replicates). G) MFI of S100A9 in splenic PMN-MDSCs (*n* = 10). H) Western blot analysis of S100A9, p-STAT6, and t-STAT6 in splenic PMN-MDSCs (replicated three times). I) Proportion of decidual (d)M-MDSCs in CD45^+^ cells (*n* = 10). Data are presented as mean ± SEM. Each dot represents a single mouse. ns, not significant; **p* < 0.05, ***p* < 0.01, ****p* < 0.001, *****p* < 0.0001. Statistical significance was determined using a Student’s *t-*test (D–G) or one-way ANOVA (B, C, and I). Post-hoc analyses were performed using Tukey’s test (B, C, and I).

**Figure S9**. No changes are observed in dPMN-MDSC levels, fetal growth parameters, or inflammation in the circulation and placental tissues of AMA mice. A–E) Comparison of dPMN-MDSC levels (A, *n* = 8, 9), placental (B) and fetal (C) weights, and crown-to-rump (D) and tail (E) lengths between male and female fetuses from AMA mice (*n* = 30 and 35, respectively). F) Secretion levels of IFN-γ, TNF-α, and IL-10 in the plasma (*n* = 4). G, H) Representative H&E staining images alongside statistical analysis of inflammatory cell infiltration among the decidua (*n* = 8, scalar bar: 50 μm). Data are presented as mean ± SEM. Each dot represents a single mouse. ns, not significant; **p* < 0.05, ***p* < 0.01, ****p* < 0.001, *****p* < 0.0001. Statistical significance was determined using a Student’s *t-*test (A−F) or Mann−Whitney test (H). D: decidua; JZ: junctional zone.

**Figure S10**. XCR1 is specifically expressed in dPMN-MDSCs. A) Percentages of XCR1^+^ cells in dPMN-MDSCs at embryonic day (E) 6.5 and E13.5 between HP and AMA mice (*n* = 4). B) Bubble diagrams depicting the expression of signature genes. C) The distribution of *Xcr1* expression among murine decidual tissues at E14. D) Bubble plots showing the expression of neutrophil and canonical DC markers in the neutrophil cluster. E, F) Representative flow cytometry plots (E) and percentages (F) of XCR1^+^ cells in different cells (*n* = 6). G) Percentages of XCR1^+^ cells in splenic PMN-MDSCs, DICs, and trophoblasts between HP and AMA mice (*n* = 6). H) mRNA expression level of *Xcl1* in PMN-MDSCs (*n* = 4 replicates). Data are presented as mean ± SEM. Each dot represents a single mouse. ns, not significant; **p* < 0.05, ***p* < 0.01, ****p* < 0.001, *****p* < 0.0001. Statistical significance was determined using a Student’s *t-*test (A, F, G, and H) or Mann−Whitney test (H).

**Figure S11**. XCL1 is specifically expressed in trophoblasts. A) Percentages of XCL1^+^ cells in trophoblasts, DICs, and DSCs (*n* = 6). B) Percentages of XCL1^+^ cells in trophoblasts at E6.5 and E13.5 (*n* = 4). Data are presented as mean ± SEM. Each dot represents a single mouse. ns, not significant; **p* < 0.05, ***p* < 0.01, ****p* < 0.001, *****p* < 0.0001. Statistical significance was determined using a Student’s *t-*test (A and B).

**Figure S12**. The expression levels of OPN, OGN, and PTN are diminished in dPMN-MDSCs of AMA mice. A) MFI of OPN, OGN, and PTN in dPMN-MDSCs from HP mice at E6.5, E13.5, and E18.5 (*n* = 4). B) mRNA expression levels of *Spp1*, *Ogn*, *Ptn*, *Vegf*, *Igf2*, and *Itgad* in dPMN-MDSCs (*n* = 4 replicates). C) MFI of OPN, OGN, and PTN in dPMN-MDSCs from HP and AMA mice (*n* = 10). D–F) MFI of OPN, OGN, and PTN in dPMN-MDSCs from AMA mice at E6.5 and E13.5 (*n* = 4). G–I) Secretion levels of OPN, OGN, and PTN in each group (*n* = 4). Data are presented as mean ± SEM. Each dot represents a single mouse. ns, not significant; **p* < 0.05, ***p* < 0.01, ****p* < 0.001, *****p* < 0.0001. Statistical significance was determined using a Student’s *t-*test (B–I), Mann−Whitney test (C), or one-way ANOVA (A). Post-hoc analyses were performed using Tukey’s test (A).

**Figure S13**. Maternal deficiency of *Xcr1* does not result in significant inflammation or increases in other DIC levels. A) Percentages of dM-MDSCs in CD45^+^ cells (*n* = 4). B, C) Proportions of splenic PMN-MDSCs (B) and M-MDSCs (C) in CD45^+^ cells (*n* = 4). D) Comparison of DIC levels in CD45^+^ cells across the indicated groups (*n* = 4). E) Secretion levels of IFN-γ, TNF-α, and IL-10 in the plasma (*n* = 4). F, G) Representative H&E staining alongside statistical analysis of inflammatory cell infiltration among the decidua (*n* = 8, scalar bar: 50 μm). H) Percentages of XCR1^+^ cells among other DICs across the indicated groups (*n* = 4). Data are presented as mean ± SEM. Each dot represents a single mouse. ns, not significant; **p* < 0.05, ***p* < 0.01, ****p* < 0.001, *****p* < 0.0001. Statistical significance was determined using Student’s *t-*test (E), Mann−Whitney test (G), or one-way ANOVA (A–D and H). Post-hoc analyses were performed using Tukey’s test (A–D and H) or Dunnett’s T3 test (D).

**Figure S14**. No significant differences in dM-MDSC, splenic PMN-MDSC, splenic M-MDSC, or XCL1^+^ trophoblast levels across different treatment groups. A) Proportions of dM-MDSCs in CD45^+^ cells across different groups (*n* = 4). B, C) Percentages of splenic PMN-MDSCs (B) and M-MDSCs (C) in CD45^+^ cells across different groups (*n* = 4). D) Proportions of dM-MDSCs in CD45^+^ cells under different treatments (*n* = 6). E, F) Percentages of splenic PMN-MDSCs (E) and M-MDSCs (F) in CD45^+^ cells under different treatments (*n* = 6). G) Percentages of XCL1^+^ cells in trophoblasts (*n* = 6). Data are presented as mean ± SEM. Each dot represents a single mouse. ns, not significant; **p* < 0.05, ***p* < 0.01, ****p* < 0.001, *****p* < 0.0001. Statistical significance was determined using a Student’s *t-*test (D–G) or one-way ANOVA (A–C). Post-hoc analyses were performed using Tukey’s test (A–C).

**Figure S15.** FOXO1 activity is significantly elevated in dPMN-MDSCs during the third trimester. A) mRNA expression levels of *FOXO3*, *FOXO4*, and *FOXO6* in dPMN-MDSCs from HP and AMA individuals (*n* = 4 replicates). B) MFI of FOXO1, phosphorylated FOXO1 (pFOXO1), and pAKT1 in the dPMN-MDSCs from HP mice at E6.5, E13.5, and E18.5 (*n* = 4). C–E) MFI of FOXO1, pFOXO1, and pAKT1 in the dPMN-MDSCs from AMA mice at E6.5 and E13.5 (*n* = 4). Data are presented as mean ± SEM. Each dot represents a single individual or mouse. ns, not significant; **p* < 0.05, ***p* < 0.01, ****p* < 0.001, *****p* < 0.0001. Statistical significance was determined using a Student’s *t-*test (A and C–E) or one-way ANOVA (B). Post-hoc analyses were performed using Tukey’s test (B).

**Figure S16**. The expression of FOXO1 in dPMN-MDSCs is crucial for fetal development. A) Representative image of the fetuses and placentas were shown. B, C) Flow cytometry plots (B) and percentages (C) of dM-MDSCs in CD45^+^ cells between the groups (*n* = 6). D–F) Flow cytometry plots (D) and proportions of splenic PMN-MDSCs (E) and M-MDSCs (F) in CD45^+^ cells (*n* = 6). G, H) Flow cytometry plots (G) and percentages (H) of XCR1^+^ cells in dPMN-MDSCs (*n* = 6). I) mRNA expression level of *Xcr1* in dPMN-MDSCs (*n* = 4 replicates). J) MFI of pAKT1 in dPMN-MDSCs (*n* = 6). K, L) mRNA expression level (K, *n =* 4 replicates) and proportion (L, *n* = 6) of XCL1^+^ cells in trophoblasts. M, N) mRNA expression level (M, *n =* 4 replicates) and MFI (N, *n* = 6) of FOXO1 in dPMN-MDSCs. Data are presented as mean ± SEM. Each dot represents a single mouse. ns, not significant; **p* < 0.05, ***p* < 0.01, ****p* < 0.001, *****p* < 0.0001. Statistical significance was determined using a Student’s *t-*test (C, E, F, and H–N) or Mann−Whitney test (M).

**Figure S17**. OXPHOS-related genes are highly expressed in decidual XCR1^+^PMN-MDSCs during the third trimester. A) Experimental flowchart of cleavage under targets and tagmentation (CUT&Tag). B, C) Signal intensity plots (B) and bar charts (C) of genomic feature distributions of FOXO1 in decidual XCR1^+^PMN-MDSCs of HP and AMA mice. D) Gene Ontology (GO) analysis of the differential targets of FOXO1-binding between HP and AMA mice. E) mRNA expression levels of *Lmna*, *Plaur*, and *Casp9* in decidual XCR1^+^PMN-MDSCs of HP mice at different gestational stages (*n* = 4 replicates). F–H) mRNA expression levels of *Lmna*, *Plaur*, and *Casp9* in decidual XCR1^+^PMN-MDSCs of AMA mice at E6.5 and E13.5 (*n* = 4 replicates). Data are presented as mean ± SEM. Each dot represents a single mouse. ns, not significant; **p* < 0.05, ***p* < 0.01, ****p* < 0.001, *****p* < 0.0001. Statistical significance was determined using a Student’s *t*-test (F–H) or one-way ANOVA (E). Post-hoc analyses were performed using Tukey’s test (E) or Dunnett’s T3 test (E).

**Figure S18**. FOXO1 interacts with the promoters of OXPHOS-related genes in murine dPMN-MDSCs. A) Western blot analysis demonstrating the expression levels of FOXO1, XCL1, and XCR1 in 293T cells following transfection (replicated twice). B) Western blot analysis revealing FOXO1 expression in 293T cells upon FOXO1 knockdown or overexpression (replicated twice). C) Identification of the FOXO1 binding motif within the promoters of *Lmna*, *Plaur*, and *Casp9*. D) The optimal binding sites located on the promoters of *Lmna*, *Plaur*, and *Casp9* (*n* = 3 replicates). Data are presented as mean ± SEM. Each dot represents a single mouse. ns, not significant; **p* < 0.05, ***p* < 0.01, ****p* < 0.001, *****p* < 0.0001. Statistical significance was determined using a Student’s *t-*test (D).

**Figure S19**. AMA and pregnant *Xcr1*^–/–^ mice exhibit metabolic imbalance in decidual XCR1^+^PMN-MDSCs. A, B) Metabolic dependency (A) and concentrations of ATP and lactate (B) in decidual XCR1^+^PMN-MDSCs between HP and AMA mice (*n* = 4). C, D) Metabolic dependency (C) and concentrations of ATP and lactate (D) in decidual XCR1^+^PMN-MDSCs between pregnant *Xcr1*^+/+^ and *Xcr1*^–/–^ mice (*n* = 4). E, F) Metabolic dependency (E) and concentrations of ATP and lactate (F) in decidual XCR1^+^PMN-MDSCs between AMA mice received either dimethyl sulfoxide (DMSO) or recombinant mouse protein XCL1 (rXCL1) (*n* = 4). G–I) Basal glycolysis and glycolytic capacities of decidual XCR1^+^PMN-MDSCs across different groups (*n* = 3 replicates). Data are presented as mean ± SEM. Each dot represents a single mouse. ns, not significant; **p* < 0.05, ***p* < 0.01, ****p* < 0.001, *****p* < 0.0001. Statistical significance was determined using a Student’s *t*-test (A–F, H, and I).

**Figure S20**. Metabolic imbalance in decidual XCR1^+^PMN-MDSCs of pregnant *Foxo1*^fl/fl^*S100a8*^cre^ mice. A) mRNA expression levels of *Lmna*, *Plaur*, and *Casp9* in decidual XCR1^+^PMN-MDSCs (*n* = 4 replicates). B) Metabolic dependency of decidual XCR1^+^PMN-MDSCs (*n* = 4). C) Concentrations of ATP and lactate in decidual XCR1^+^PMN-MDSCs (*n* = 4). D–F) Basal glycolysis and glycolytic capacities of decidual XCR1^+^PMN-MDSCs (*n* = 3 replicates). Data are presented as mean ± SEM. Each dot represents a single mouse. ns, not significant; **p* < 0.05, ***p* < 0.01, ****p* < 0.001, *****p* < 0.0001. Statistical significance was determined using a Student’s *t*-test (A–C, E, and F) or Mann−Whitney test (A and C).

**Figure S21**. Overexpression of XCL1 in trophoblasts and XCR1 or FOXO1 in dPMN-MDSCs. A) Representative fluorescence images of trophoblasts after transfection with either adenoviral control vectors (AdV-Ctrl) or AdV-XCL1. B) Transfection efficiency for AdV-Ctrl and AdV-XCL1 in trophoblasts (*n* = 6). C) mRNA expression level of *Xcl1* in trophoblasts (*n* = 4 replicates). D) Percentages of XCL1^+^ cells in trophoblasts (*n* = 4). E) Secretion levels of XCL1 in the supernatant from trophoblast cultures (*n* = 4). F) Representative fluorescence images of primary dPMN-MDSCs after transfection with either AdV-Ctrl or AdV-XCR1. G) Transfection efficiency for AdV-Ctrl and AdV-XCR1 in dPMN-MDSCs (*n* = 6). H) mRNA expression level of *Xcr1* in dPMN-MDSCs (*n* = 4 replicates). I) Percentages of XCR1^+^ cells in dPMN-MDSCs (*n* = 4). J) Representative fluorescence images of primary dPMN-MDSCs after transfection with either AdV-Ctrl or AdV-FOXO1. K) Transfection efficiency for AdV-Ctrl and AdV-FOXO1 in dPMN-MDSCs (*n* = 6). L, M) mRNA expression level (L) and MFI (M) of FOXO1 in dPMN-MDSCs (*n* = 4). Data are presented as mean ± SEM. Each dot represents a single mouse. ns, not significant; **p* < 0.05, ***p* < 0.01, ****p* < 0.001, *****p* < 0.0001. Statistical significance was determined using a Student’s *t*-test (B, D, E, G, I, K, and M) or Mann−Whitney test (C, H, and L).

**Figure S22**. Oltipraz treatment and cell-transfer don’t increase other DIC levels but enhance mitochondrial activity in decidual XCR1^+^PMN-MDSCs of AMA mice. A) Proportions of dM-MDSCs in CD45^+^ cells (*n* = 6, 6, and 4, respectively). B, C) Percentages of splenic PMN-MDSCs (B) and M-MDSCs (C) in CD45^+^ cells (*n* = 6, 6, and 4, respectively). D) Percentages of various DICs under different treatments (*n* = 4). E) Percentages of XCR1^+^ cells in various DICs (*n* = 4). F) mRNA expression levels of *Foxo1* in dPMN-MDSCs (*n* = 4 replicates). G) Metabolic dependency of decidual XCR1^+^PMN-MDSCs (*n* = 4). H) Concentrations of ATP and lactate in decidual XCR1^+^PMN-MDSCs (*n* = 4). I) mRNA expression levels of nutrient transporters in dPMN-MDSCs (*n* = 4 replicates). J, K) mRNA expression level (J, *n =* 4 replicates) and percentage (K, *n =* 6) of XCL1^+^ in trophoblasts. Data are presented as mean ± SEM. Each dot represents a single mouse. ns, not significant; **p* < 0.05, ***p* < 0.01, ****p* < 0.001, *****p* < 0.0001. Statistical significance was determined using one-way ANOVA (A–K). Post-hoc analyses were performed using Tukey’s test (A–K) or Dunnett’s test (D).

**Figure S23.** Oltipraz treatment and cell-transfer don’t increase other DIC levels and slightly alter mitochondrial activity in dPMN-MDSCs of pregnant *Xcr1*^−/−^ mice. A) Proportions of dM-MDSCs in CD45^+^ cells (*n* = 4). B, C) Percentages of splenic PMN-MDSCs (B) and M-MDSCs (C) in CD45^+^ cells (*n* = 4). D) Percentages of various DICs with different treatments (*n* = 4). E) Percentages of XCR1^+^ cells in various DICs (*n* = 4). F) mRNA expression levels of *Foxo1* in dPMN-MDSCs (*n* = 4 replicates). G) Metabolic dependency of dPMN-MDSCs (*n* = 4). H) Concentrations of ATP and lactate in dPMN-MDSCs (*n* = 4). I) mRNA expression levels of nutrient transporters in dPMN-MDSCs (*n* = 4 replicates). J, K) mRNA level (J, *n =* 4 replicates) and percentage (K, *n =* 4) of XCL1^+^ in trophoblasts. Data are presented as mean ± SEM. Each dot represents a single mouse. ns, not significant; **p* < 0.05, ***p* < 0.01, ****p* < 0.001, *****p* < 0.0001. Statistical significance was determined using one-way ANOVA (A–K). Post-hoc analyses were performed using Tukey’s test (A–K) or Dunnett’s test (E).

**Figure S24.** Decidual XCR1^+^PMN-MDSCs from AMA patients exhibit metabolic imbalance. A) The FOXO1 binding motif located on the promoters of *Lmna*, *Plaur*, and *Casp9*. B) The optimal binding sites for the *LMNA*, *PLAUR*, and *CASP9* promoters (*n* = 3 replicates). C) Metabolic dependency of decidual XCR1^+^PMN-MDSCs (*n* = 6). D) Concentrations of ATP and lactate in decidual XCR1^+^PMN-MDSCs (*n* = 4). E–G) Basal glycolysis and glycolytic capacities of decidual XCR1^+^PMN-MDSCs (*n* = 3 replicates). Data are presented as mean ± SEM. Each dot represents a single individual. ns, not significant; **p* < 0.05, ***p* < 0.01, ****p* < 0.001, *****p* < 0.0001. Statistical significance was determined using a Student’s *t-*test (B–D, F, and G) or Mann−Whitney test (D).

**Figure S25.** The XCL1–XCR1 axis mediates the crosstalk between trophoblasts and dPMN-MDSCs, thereby promoting fetal growth. Disruption of this axis impairs FOXO1 activity and leads to metabolic imbalance in decidual XCR1^+^PMN-MDSCs, contributing to fetal growth restriction in AMA conditions.

**Figure S26.** Assumptions of Clinical Data Models. A–D) Multicollinearity was evaluated using the variance inflation factor for all confounding variables. The fundamental model assumptions—linearity, independence, normality, and homoscedasticity—were assessed using residuals against fitted values (scatter plot), normal Q–Q plots, and a scale-location plots. The sample sizes were as follows: HP (*n* = 23), AMA (*n* = 28).

**Figure S27.** The gating strategy of human samples.

**Figure S28.** The gating strategy of murine samples.

**Figure S29.** The purified efficiency of dPMN-MDSCs and trophoblasts in both human and mouse models.

**Table S1. Clinical characteristics of the HP and AMA cohorts used for scRNA-sequencing**

| **Sample** | **Maternal age (years)** | **Gestational age (weeks)** | **Number of conception (s)** | **Number of production (s)** | **Height (cm)** | **Weight (kg)** | **BMI (kg/m^2^)** |
| --- | --- | --- | --- | --- | --- | --- | --- |
| HP_1 | 28 | 39+0 | 1 | 0 | 150 | 65.0 | 28.9 |
| HP_2 | 27 | 38+4 | 2 | 1 | 162 | 77.0 | 29.3 |
| HP_3 | 28 | 38+1 | 1 | 0 | 158 | 58.0 | 23.2 |
| HP_4 | 26 | 39+0 | 1 | 0 | 161 | 58.4 | 22.5 |
| AMA_1 | 40 | 38+4 | 3 | 2 | 160 | 58.5 | 22.9 |
| AMA_2 | 38 | 38+6 | 2 | 1 | 155 | 70.0 | 29.1 |
| AMA_3 | 40 | 38+6 | 2 | 0 | 158 | 60.7 | 24.3 |

The HP and AMA cohorts contained pregnant women who received a cesarean section due to breech presentation, previous caesarean section, placenta previa, and incompetent cervix.

**Table S2. Clinical characteristics of the HP and AMA cohorts used for flow cytometry and clinical relevance**

| **Characteristics** | **HP (*n* = 23)** | **AMA (*n* = 28)** |
| --- | --- | --- |
| Maternal age (years) | 28.74 ± 2.39******** | 38.57 ± 2.42 |
| Gestational age (weeks) | 38.43 ± 0.84 | 38.18 ± 0.98 |
| Number of conception (s) | 1.73 ± 1.05**^###^** | 3.39 ± 2.24 |
| Number of production (s) | 0.69 ± 0.87 | 0.92 ± 0.97 |
| Height (cm) | 158.40 ± 4.75 | 157.60 ± 6.29 |
| Weight (kg) | 62.45 ± 7.90 | 64.82 ± 8.13 |
| BMI (kg/m^2^) | 24.93 ± 2.91 | 26.03 ± 2.40 |

The HP and AMA cohorts contained pregnant women who received a cesarean section due to breech presentation, previous caesarean section, placenta previa, and incompetent cervix. Data are presented as mean ± standard deviation (SD). **p* < 0.05, using a Student’s *t-*test. ^#^*p* < 0.05, using a Mann–Whitney test.

**Table S3. Independent variables of HP used for multivariate linear regression analysis**

| **Maternal age** | 26 | | 30 | 28 | 29 | 31 | 29 | 33 | 30 | 32 | 29 | 30 | 33 | 24 | 29 | 28 | 30 | 29 | 26 | 27 | 30 | 25 | 27 | 26 |
| --- | --- | --- | --- | --- | --- | --- | --- | --- | --- | --- | --- | --- | --- | --- | --- | --- | --- | --- | --- | --- | --- | --- | --- | --- |
| **Gestational age** | 39 | | 38 | 39 | 38 | 39 | 39 | 38 | 39 | 39 | 39 | 37 | 37 | 37 | 38 | 39 | 37 | 39 | 39 | 38 | 39 | 40 | 38 | 39 |
| **BMI** | 22.5 | | 22.3 | 24.3 | 22.9 | 23.4 | 29.0 | 25.6 | 25.9 | 26.1 | 21.5 | 23.1 | 27.5 | 19.1 | 23.3 | 28.5 | 29.6 | 26.7 | 22.5 | 22.7 | 27.5 | 27.9 | 22.3 | 29.3 |
| **SBP** | 108 | 115 | | 117 | 106 | 105 | 105 | 108 | 106 | 112 | 111 | 106 | 111 | 118 | 117 | 109 | 112 | 109 | 109 | 98 | 109 | 106 | 111 | 129 |
| **DBP** | 58 | | 81 | 64 | 65 | 60 | 77 | 67 | 66 | 73 | 70 | 72 | 74 | 64 | 67 | 75 | 67 | 60 | 62 | 64 | 75 | 68 | 70 | 84 |
| **FPG** | 4.04 | | 4.70 | 4.20 | 3.79 | 3.89 | 3.14 | 3.55 | 3.55 | 4.19 | 4.27 | 3.85 | 4.44 | 3.68 | 4.45 | 4.12 | 3.70 | 4.14 | 4.04 | 4.12 | 3.95 | 4.06 | 4.19 | 3.37 |
| **HbA1c** | 5.6 | | 5.2 | 5.2 | 4.9 | 5.3 | 5.0 | 6.0 | 4.8 | 4.6 | 5.9 | 5.2 | 5.0 | 4.1 | 5.8 | 4.3 | 6.4 | 5.9 | 5.6 | 5.4 | 4.8 | 5.3 | 4.2 | 5.5 |
| **OGTT** | 4.75 | 4.76 | | 6.46 | 6.18 | 6.31 | 5.57 | 5.82 | 6.63 | 5.94 | 5.41 | 5.85 | 6.61 | 5.88 | 5.99 | 6.26 | 6.52 | 6.67 | 4.32 | 6.08 | 5.85 | 5.69 | 5.41 | 6.84 |
| **TG** | 113 | 130 | | 150 | 104 | 81 | 123 | 135 | 98 | 152 | 110 | 138 | 105 | 142 | 126 | 102 | 204 | 109 | 156 | 132 | 96 | 108 | 68 | 123 |
| **CHOL** | 181 | 207 | | 148 | 124 | 160 | 154 | 165 | 180 | 128 | 135 | 188 | 243 | 123 | 125 | 265 | 147 | 162 | 168 | 135 | 168 | 134 | 145 | 156 |
| **HDL-C** | 77 | 74 | | 78 | 72 | 70 | 68 | 63 | 67 | 82 | 70 | 72 | 71 | 68 | 60 | 79 | 65 | 88 | 67 | 77 | 88 | 73 | 99 | 78 |
| **LDL-C** | 93 | 138 | | 104 | 143 | 80 | 99 | 102 | 115 | 108 | 96 | 82 | 145 | 150 | 100 | 152 | 102 | 98 | 75 | 100 | 79 | 91 | 78 | 105 |
| **VLDL-C** | 12 | 28 | | 32 | 37 | 19 | 21 | 23 | 20 | 25 | 22 | 15 | 12 | 32 | 28 | 38 | 10 | 11 | 22 | 18 | 20 | 21 | 38 | 27 |
| **CRP** | 2.54 | 2.10 | | 3.10 | 1.22 | 2.22 | 0.58 | 1.10 | 2.35 | 0.06 | 0.28 | 1.25 | 1.11 | 5.84 | 3.25 | 2.20 | 1.63 | 1.59 | 0.52 | 1.11 | 0.52 | 1.35 | 0.55 | 1.23 |
| **WBC** | 11.89 | 7.58 | | 10.20 | 8.27 | 8.62 | 5.63 | 8.69 | 10.41 | 7.33 | 9.60 | 6.05 | 6.45 | 6.82 | 5.21 | 7.57 | 11.64 | 8.61 | 7.55 | 9.55 | 11.13 | 7.26 | 8.45 | 10.27 |
| **Neu** | 5.13 | 7.87 | | 5.78 | 6.81 | 3.83 | 6.78 | 5.40 | 3.69 | 4.73 | 3.83 | 5.89 | 6.73 | 4.73 | 5.75 | 5.31 | 5.38 | 6.65 | 6.00 | 7.09 | 8.06 | 7.17 | 6.19 | 7.87 |

**Table S4. Independent variables of AMA used for multivariate linear regression analysis**

| **Maternal age** | 38 | 35 | 40 | 35 | 38 | 46 | 41 | 41 | 40 | 37 | 40 | 38 | 37 | 42 | 39 | 36 | 39 | 36 | 38 | 37 | 37 | 36 | 36 | 41 | 40 | 36 | 40 | 39 |
| --- | --- | --- | --- | --- | --- | --- | --- | --- | --- | --- | --- | --- | --- | --- | --- | --- | --- | --- | --- | --- | --- | --- | --- | --- | --- | --- | --- | --- |
| **Gestational age** | 39 | 40 | 39 | 38 | 38 | 39 | 37 | 39 | 39 | 38 | 38 | 38 | 38 | 38 | 38 | 37 | 39 | 39 | 36 | 38 | 38 | 38 | 40 | 36 | 38 | 39 | 36 | 38 |
| **BMI** | 25.5 | 23.4 | 25.6 | 27.0 | 28.5 | 24.5 | 25.0 | 26.4 | 22.9 | 24.8 | 29.1 | 29.7 | 25.1 | 28.4 | 29.6 | 28.9 | 29.3 | 24.3 | 25.8 | 29.7 | 22.3 | 25.8 | 23.1 | 22.3 | 24.7 | 23.0 | 27.0 | 27.1 |
| **SBP** | 114 | 128 | 115 | 117 | 96 | 111 | 131 | 118 | 128 | 124 | 106 | 121 | 119 | 117 | 116 | 109 | 101 | 112 | 116 | 123 | 114 | 114 | 128 | 115 | 117 | 96 | 111 | 116 |
| **DBP** | 86 | 84 | 73 | 74 | 54 | 69 | 77 | 81 | 80 | 66 | 71 | 82 | 60 | 66 | 76 | 64 | 66 | 74 | 60 | 81 | 66 | 86 | 84 | 73 | 74 | 54 | 69 | 60 |
| **FPG** | 4.50 | 3.85 | 4.61 | 3.96 | 3.99 | 4.31 | 4.02 | 4.00 | 3.87 | 4.81 | 3.71 | 4.70 | 4.81 | 4.10 | 3.70 | 4.95 | 4.33 | 3.32 | 4.83 | 4.63 | 5.10 | 3.99 | 3.86 | 4.31 | 4.96 | 4.10 | 3.78 | 4.69 |
| **HbA1c** | 5.0 | 5.7 | 5.3 | 5.1 | 5.6 | 6.0 | 4.9 | 5.3 | 5.3 | 5.8 | 4.7 | 5.6 | 6.1 | 5.1 | 5.0 | 5.7 | 5.2 | 4.8 | 4.8 | 5.1 | 5.6 | 4.7 | 4.9 | 4.5 | 5.2 | 5.1 | 4.2 | 6.0 |
| **OGTT** | 7.23 | 6.67 | 5.67 | 7.93 | 4.19 | 4.96 | 6.56 | 6.69 | 8.42 | 6.97 | 7.77 | 5.69 | 6.54 | 5.99 | 6.50 | 6.21 | 4.70 | 4.80 | 5.05 | 6.29 | 5.35 | 4.25 | 5.85 | 6.20 | 6.12 | 5.99 | 4.82 | 5.38 |
| **TG** | 209 | 175 | 198 | 220 | 105 | 121 | 196 | 130 | 165 | 145 | 131 | 115 | 121 | 105 | 170 | 168 | 156 | 142 | 105 | 165 | 133 | 125 | 100 | 169 | 174 | 135 | 146 | 174 |
| **CHOL** | 261 | 210 | 159 | 109 | 278 | 150 | 250 | 238 | 156 | 128 | 167 | 145 | 205 | 238 | 199 | 165 | 200 | 138 | 147 | 222 | 232 | 235 | 112 | 258 | 268 | 240 | 281 | 156 |
| **HDL-C** | 83 | 75 | 32 | 93 | 63 | 58 | 70 | 72 | 69 | 92 | 60 | 77 | 72 | 81 | 102 | 66 | 79 | 71 | 77 | 63 | 82 | 68 | 72 | 99 | 85 | 66 | 84 | 69 |
| **LDL-C** | 111 | 100 | 92 | 88 | 103 | 102 | 75 | 66 | 82 | 107 | 135 | 111 | 90 | 68 | 71 | 132 | 90 | 114 | 100 | 102 | 121 | 128 | 131 | 100 | 112 | 132 | 85 | 96 |
| **VLDL-C** | 33 | 19 | 23 | 44 | 35 | 50 | 32 | 28 | 51 | 20 | 42 | 38 | 26 | 30 | 36 | 49 | 51 | 36 | 40 | 31 | 25 | 35 | 40 | 26 | 20 | 44 | 52 | 53 |
| **CRP** | 3.71 | 0.00 | 4.27 | 0.00 | 1.75 | 0.50 | 1.06 | 2.55 | 4.25 | 0.50 | 0.60 | 0.00 | 1.00 | 6.20 | 5.89 | 0.00 | 1.26 | 1.03 | 3.29 | 1.50 | 2.94 | 0.00 | 0.52 | 0.50 | 1.05 | 1.68 | 0.00 | 2.12 |
| **WBC** | 8.45 | 9.97 | 8.75 | 7.44 | 9.53 | 6.58 | 8.60 | 5.23 | 8.72 | 4.10 | 8.21 | 8.72 | 5.65 | 14.00 | 6.42 | 8.76 | 9.33 | 9.01 | 8.92 | 8.93 | 6.47 | 6.99 | 6.47 | 10.29 | 7.52 | 10.31 | 8.50 | 9.80 |
| **Neu** | 5.17 | 5.37 | 6.90 | 5.99 | 6.18 | 5.97 | 6.80 | 4.74 | 6.57 | 3.19 | 5.82 | 2.81 | 4.85 | 4.31 | 4.89 | 5.05 | 6.59 | 4.15 | 6.00 | 6.34 | 1.03 | 6.09 | 7.14 | 5.66 | 4.89 | 5.17 | 7.15 | 6.05 |

**Table S5. The multivariate linear regression analysis of the HP and AMA cohorts**

|  | **HP (*n* = 23)** | | | **AMA (*n* = 28)** | | |
| --- | --- | --- | --- | --- | --- | --- |
|  | **β** | **95% CI** | ***P*** | **β** | **95% CI** | ***P*** |
| Maternal age | -0.39 | (-1.08, 0.29) | 0.2083 | -1.14 | (-1.99, -0.29) | *0.0124* |
| Gestational weeks (ref = 38–41) | 1.67 | (-0.36, 3.70) | 0.0912 | -0.76 | (-3.70, 2.17) | 0.5778 |
| BMI (ref = 24.0–30.0 kg/m^2^) | -0.48 | (-1.32, 0.34) | 0.2002 | -0.26 | (-1.10, 0.56) | 0.4933 |
| SBP (ref = 90–139 mmHg) | 0.07 | (-0.22, 0.36) | 0.5868 | -0.12 | (-0.51, 0.26) | 0.4993 |
| DBP (ref = 60–89 mmHg) | -0.05 | (-0.38, 0.27) | 0.7049 | 0.20 | (-0.15, 0.55) | 0.2382 |
| FPG (ref = 3.6–5.1 mmol/L) | 0.05 | (-4.39, 4.50) | 0.9757 | 0.22 | (-5.29, 5.74) | 0.9293 |
| HbA1c (ref = 4.0–6.0%) | -1.13 | (-5.09, 2.82) | 0.5095 | -0.47 | (-8.16, 7.20) | 0.8938 |
| OGTT (ref = 3.9–7.8 mmol/L) | 0.78 | (-2.02, 3.59) | 0.5184 | 0.14 | (-1.88, 2.17) | 0.8783 |
| TG (ref = 0–200 mg/dL) | 0.04 | (-0.01, 0.10) | 0.0928 | -0.02 | (-0.07, 0.01) | 0.1654 |
| CHOL (ref = 0–280 mg/dL) | 0.03 | (-0.01, 0.07) | 0.0769 | -0.01 | (-0.05, 0.01) | 0.2456 |
| HDL-C (ref = 40–100 mg/dL) | -0.02 | (-0.27, 0.22) | 0.8246 | -0.08 | (-0.24, 0.08) | 0.3080 |
| LDL-C (ref = 0–130 mg/dL) | -0.05 | (-0.12, 0.02) | 0.1245 | -0.11 | (-0.27, 0.05) | 0.1615 |
| VLDL-C (ref = 12–52 mg/dL) | 0.04 | (-0.15, 0.23) | 0.6192 | 0.01 | (-0.19, 0.22) | 0.8768 |
| CRP (ref = 0.0–6.0 mg/dL) | -0.37 | (-2.06, 1.31) | 0.6075 | -0.18 | (-1.44, 1.06) | 0.7467 |
| WBC (ref = 3.0–12.0 *10^9^/L) | 0.42 | (-0.34, 1.18) | 0.2281 | -0.05 | (-1.16, 1.04) | 0.9111 |
| Neu (ref = 1.80-6.30 *10^9^/L) | 1.02 | (-0.25, 2.30) | 0.0984 | -0.80 | (-2.59, 0.98) | 0.3453 |

BMI: Body Mass Index; SBP: Systolic Blood Pressure; DBP: Diastolic Blood Pressure; FPG: Fasting Plasma Glucose; HbA1c: Glycated Hemoglobin A1c; OGTT: Oral Glucose Tolerance Test; TG: Triglyceride; CHOL: Cholesterol; HDL-C: High-Density Lipoprotein Cholesterol; LDL-C: Low-Density Lipoprotein Cholesterol; VLDL-C: Very Low-Density Lipoprotein Cholesterol; CRP: C-Reactive Protein; WBC: White Blood Cell; Neu: Neutrophils. 95% CI: 95% Confidence Interval (lower–upper). Values in italics are significant after adjusting for multiple comparison.

**Table S6. The VIF of the HP cohort**

| **Variables** | **VIF value** | **Status** |
| --- | --- | --- |
| WBC | 1.9207 | Acceptable multicollinearity |
| CHOL | 2.1574 | Acceptable multicollinearity |
| TG | 2.3334 | Acceptable multicollinearity |
| FPG | 2.4253 | Acceptable multicollinearity |
| Neu | 2.4659 | Acceptable multicollinearity |
| Maternal age | 2.5194 | Acceptable multicollinearity |
| VLDL-C | 2.5351 | Acceptable multicollinearity |
| Gestational age | 2.7076 | Acceptable multicollinearity |
| LDL-C | 2.8149 | Acceptable multicollinearity |
| SBP | 3.1002 | Acceptable multicollinearity |
| OGTT-2h | 3.1031 | Acceptable multicollinearity |
| CRP | 4.2064 | Acceptable multicollinearity |
| DBP | 4.5077 | Acceptable multicollinearity |
| HDL-C | 4.5725 | Acceptable multicollinearity |
| HbA1c | 5.0114 | Moderate multicollinearity |
| BMI | 5.4318 | Moderate multicollinearity |

BMI: Body Mass Index; SBP: Systolic Blood Pressure; DBP: Diastolic Blood Pressure; FPG: Fasting Plasma Glucose; HbA1c: Glycated Hemoglobin A1c; OGTT: Oral Glucose Tolerance Test; TG: Triglyceride; CHOL: Cholesterol; HDL-C: High-Density Lipoprotein Cholesterol; LDL-C: Low-Density Lipoprotein Cholesterol; VLDL-C: Very Low-Density Lipoprotein Cholesterol; CRP: C-Reactive Protein; WBC: White Blood Cell; Neu: Neutrophils.

**Table S7. Model assumption statistics for the HP cohort**

| **Statistic** | **Value** |
| --- | --- |
| Multiple R | 0.9316 |
| R Square | 0.8680 |
| Adjusted R Square | 0.5159 |
| Standard Error | 1.9989 |
| Observations | 23 |

**Table S8. The VIF of the AMA cohort**

| **Variables** | **VIF value** | **Status** |
| --- | --- | --- |
| BMI | 1.9643 | Acceptable multicollinearity |
| Maternal age | 2.1196 | Acceptable multicollinearity |
| WBC | 2.1541 | Acceptable multicollinearity |
| TG | 2.1627 | Acceptable multicollinearity |
| OGTT-2h | 2.2909 | Acceptable multicollinearity |
| CRP | 2.4370 | Acceptable multicollinearity |
| VLDL-C | 2.4570 | Acceptable multicollinearity |
| HDL-C | 2.6471 | Acceptable multicollinearity |
| CHOL | 2.7757 | Acceptable multicollinearity |
| Neu | 2.8849 | Acceptable multicollinearity |
| FPG | 3.2342 | Acceptable multicollinearity |
| Gestational age | 4.5692 | Acceptable multicollinearity |
| LDL-C | 5.2230 | Moderate multicollinearity |
| DBP | 5.3296 | Moderate multicollinearity |
| SBP | 5.6706 | Moderate multicollinearity |
| HbA1c | 6.3572 | Moderate multicollinearity |

BMI: Body Mass Index; SBP: Systolic Blood Pressure; DBP: Diastolic Blood Pressure; FPG: Fasting Plasma Glucose; HbA1c: Glycated Hemoglobin A1c; OGTT: Oral Glucose Tolerance Test; TG: Triglyceride; CHOL: Cholesterol; HDL-C: High-Density Lipoprotein Cholesterol; LDL-C: Low-Density Lipoprotein Cholesterol; VLDL-C: Very Low-Density Lipoprotein Cholesterol; CRP: C-Reactive Protein; WBC: White Blood Cell; Neu: Neutrophils.

**Table S9. Model assumption statistics for the AMA cohort**

| **Statistic** | **Value** |
| --- | --- |
| Multiple R | 0.7662 |
| R Square | 0.5871 |
| Adjusted R Square | 0.0135 |
| Standard Error | 3.3921 |
| Observations | 28 |

**Table S10. List of antibodies and reagents used in this study**

| **Antibodies and Reagents** | **Source** | **Identifier** |
| --- | --- | --- |
| **Antibodies** | | |
| PerCP-Cy5.5 anti-human CD45 | eBioscience | Cat#45-0459-42; RRID:AB_10717530 |
| PE-Cy7 anti-human CD14 | eBioscience | Cat#25-0149-42; RRID:AB_1582276 |
| eF450 anti-human CD15 | eBioscience | Cat#48-0159-42; RRID:AB_2016661 |
| PE anti-human LOX-1 | BioLegend | Cat#358604; RRID:AB_2562181 |
| APC anti-human LOX-1 | BioLegend | Cat#358606; RRID:AB_2563473 |
| FITC anti-human XCR1 | BioLegend | Cat#372611; RRID:AB_2715830 |
| eF450 anti-human CD3 | eBioscience | Cat#48-0037-42; RRID:AB_1272055 |
| Biotin anti-human CD3 | BioLegend | Cat#300404; RRID:AB_314058 |
| Biotin anti-human CD14 | eBioscience | Cat#13-0149-82; RRID:AB_466373 |
| APC anti-human HLA-DR | eBioscience | Cat#17-9956-42; RRID:AB_10670347 |
| Biotin anti-human CD19 | BioLegend | Cat#302204; RRID:AB_314234 |
| Biotin anti-human CD20 | ThermoFisher | Cat#MA1-19525; RRID:AB_1071557 |
| Biotin anti-human CD56 | eBioscience | Cat#13-0567-82; RRID:AB_10596499 |
| Biotin anti-human CD123 | eBioscience | Cat#13-1239-82; RRID:AB_466586 |
| eF450 anti-human CD11c | eBioscience | Cat#48-0116-41; RRID:AB_11218498 |
| PE-Cy7 anti-human CD141 | BioLegend | Cat#344109; RRID:AB_2561622 |
| APC anti-human CD206 | BioLegend | Cat#321109; RRID:AB_571884 |
| APC anti-human CD49a | BioLegend | Cat#328313; RRID:AB_2566377 |
| PE-Cy7 anti-human CD4 | TONBO | Cat#60-0049; RRID:AB_2621831 |
| PE anti-human CD4 | eBioscience | Cat#12-0048-42; RRID:AB_2016675 |
| APC anti-human CD8a | BioLegend | Cat#301049; RRID:AB_2562054 |
| PerCP-Cy5.5 anti-human CD8a | eBioscience | Cat#45-0088-42; RRID:AB_1582255 |
| Biotin anti-mouse/human CD45R/B220 | BioLegend | Cat#RA3-6B2; RRID:AB_312988 |
| Biotin anti-human CD56 (NCAM) | eBioscience | Cat#13-0567-82; RRID:AB_10596499 |
| Biotin anti-human CD11b | BioLegend | Cat#301304; RRID:AB_314156 |
| Biotin anti-human TCRγ/δ | BioLegend | Cat#331206; RRID:AB_1089216 |
| Biotin anti-human TCRα/β | eBioscience | Cat#13-9986-82; RRID:AB_467025 |
| Biotin anti-human CD14 | BioLegend | Cat#367106; RRID:AB_2566618 |
| Biotin anti-human CD34 | BioLegend | Cat#343524; RRID:AB_10900977 |
| ***Continued*** |  |  |
| Biotin anti-human CD19 | BioLegend | Cat#302204; RRID:AB_314234 |
| Biotin anti-human CD3 | BioLegend | Cat#300404; RRID:AB_314058 |
| Biotin anti-human CD123 | eBioscience | Cat#13-1239-82; RRID:AB_466586 |
| Biotin anti-human CD11c | eBioscience | Cat#13-0116-82; RRID:AB_466366 |
| Biotin anti-human CD8a | BioLegend | Cat#301004; RRID:AB_314122 |
| Biotin anti-human FceR1 alpha | eBioscience | Cat#13-5899-82; RRID:AB_466786 |
| AF647 anti-human CD370 (Clec9A) | BD Biosciences | Cat#564267; RRID:AB_2738717 |
| FITC anti-human CD127 (IL-7Rα) | BioLegend | Cat#351312; RRID:AB_10897643 |
| PE-Cy7 anti-human CD161 | eBioscience | Cat#25-1619-42; RRID:AB_10807086 |
| BV 605 anti-human CD294 (CRTH2) | BioLegend | Cat#350122; RRID:AB_2566760 |
| PB anti-human CD117 (c-Kit) | BioLegend | Cat#375210 |
| Biotin anti-human CD31 | BioLegend | Cat#102404; RRID:AB_312899 |
| PE anti-human HLA-G | eBioscience | Cat#MA1-10369; RRID:AB_11152968 |
| Biotin anti-mouse CD4 | BioLegend | Cat#100508; RRID:AB_312711 |
| Biotin anti-mouse CD8a | BioLegend | Cat#100704; RRID:AB_312743 |
| Biotin anti-mouse TER-119 | BioLegend | Cat#116204; RRID:AB_313705 |
| Biotin anti-mouse CD11c | BioLegend | Cat#117304; RRID:AB_313773 |
| Biotin anti-mouse NK1.1 | BioLegend | Cat#108704; RRID:AB_313391 |
| Biotin anti-mouse TCRγ/δ | eBioscience | Cat#13-5711-85; RRID:AB_466669 |
| Biotin anti-mouse CD5 | eBioscience | Cat#13-0051-85; RRID:AB_466340 |
| Biotin anti-mouse TCR β chain | eBioscience | Cat#13-5961-85; RRID:AB_466820 |
| Biotin anti-mouse CD3e | eBioscience | Cat#13-0031-86; RRID:AB_466321 |
| Biotin anti-mouse Ly-6G | eBioscience | Cat#13-5931-86; RRID:AB_466802 |
| Biotin anti-mouse CD11b | eBioscience | Cat#13-0112-86; RRID:AB_466361 |
| APC anti-T-bet | Biolegend | Cat#644814; RRID:AB_10901173 |
| PE anti-mouse MHCII | eBioscience | Cat#12-5322-81; RRID:AB_465930 |
| PE-Cy7 anti-mouse CD170 (Siglec F) | eBioscience | Cat#25-1702-82; RRID:AB_2802251 |
| AF647 anti-mouse CD370 (Clec9A) | Biolegend | Cat#143506; RRID:AB_2566380 |
| PB anti-mouse CD11b | BioLegend | Cat#101224; RRID:AB_755986 |
| PE-Cy7 anti-mouse Ly-6G | eBioscience | Cat#25-9668-82; RRID:AB_2811793 |
| Percp-Cy5.5 anti-mouse Ly-6C | eBioscience | Cat#45-5932-82; RRID:AB_2723343 |
| ***Continued*** | | |
| PE anti-mouse XCR1 | BioLegend | Cat#148203; RRID:AB_2563842 |
| Biotin anti- mouse CD3e | BioLegend | Cat#13-0031-86; RRID:AB_466321 |
| Biotin anti-mouse Ly-6G | BioLegend | Cat#127604; RRID:AB_1186108 |
| PE-Cy7 anti-mouse CD19 | eBioscience | Cat#25-0193-82; RRID: AB_657663 |
| Biotin anti-mouse NK1.1 | BioLegend | Cat#108704; RRID:AB_313391 |
| eF450 anti-mouse F4/80 | eBioscience | Cat#48-4801-82; RRID:AB_1548747 |
| Biotin anti-mouse CD11b | eBioscience | Cat#13-0112-86; RRID:AB_466361 |
| PE anti-mouse MHCII | eBioscience | Cat#12-5322-81; RRID:AB_465930 |
| Biotin anti-mouse CD11c | BioLegend | Cat#117304; RRID:AB_313773 |
| APC anti-mouse CD103 | eBioscience | Cat#17-1031-80; RRID:AB_1106993 |
| PE-eF610 anti-mouse CD45 | eBioscience | Cat#61-0451-82; RRID:AB_2574555 |
| Alexa Fluor647 anti-mouse PTN (H-6) | Santa Cruz | Cat#sc-74443; RRID:AB_1128556 |
| Alexa Fluor647 anti-mouse FKHR(C-9) | Santa Cruz | Cat#sc-374427; RRID:AB_10987717 |
| Alexa Fluor647 anti-mouse lymphotactin (E12) | Santa Cruz | Cat#sc-514972 AF647 |
| Mouse anti-Lymphotactin (E-12) | Santa Cruz | Cat#sc-514972 |
| CruzFluor 647 Mouse lgGk BP | Santa Cruz | Cat#sc-516179 |
| Rabbit polyclonal anti-Cytokeratin 7 | Proteintech | Cat#15539-1-AP; RRID:AB_2249769 |
| Cytokeratin 7 monoclonal antibody | eBioscience | Cat#MA1-06315 |
| PE Donkey anti-Rabbit lgG | BioLegend | Cat#406421; RRID:AB_2563484 |
| Goat anti-Rabbit IgG H&L | Abcam | Cat#ab155700 |
| APC-eFluor780 STREPTAVIDIN | ThermoFisher | Cat#47-4317-82; RRID:AB_10366688 |
| Ghost Dye Violet 510 | TONBO | Cat#13-0870 |
| Rabbit monoclonal anti-S100A9 | Cell Signaling Technology | Cat#73425; RRID:AB_2799839 |
| Rabbit monoclonal anti-Phospho-Stat6 | Cell Signaling Technology | Cat#56554; RRID:AB_2799514 |
| Rabbit monoclonal anti-FoxO1 | Cell Signaling Technology | Cat#2880; RRID:AB_2106495 |
| Normal Rabbit IgG | Cell Signaling Technology | Cat#2729; RRID:AB_1031062 |
| Rabbit polyclonal anti-beta-Tubulin | Cell Signaling Technology | Cat#2146; RRID:AB_2210545 |
| Rabbit polyclonal anti-Stat6 | Cell Signaling Technology | Cat#9362; RRID:AB_2271211 |
| Rabbit polyclonal anti-Phospho-FOXO1 | Zen Bioscience | Cat#310198 |
| Rabbit polyclonal anti-GAPDH | Zen Bioscience | Cat#380626 |
| Rabbit polyclonal anti-Phospho-AKT(Ser473) | Zen Bioscience | Cat#381555 |
| ***Continued*** | | |
| Rabbit polyclonal anti-AKT1 | Zen Bioscience | Cat#380617 |
| Rabbit polyclonal anti-CD31 | Zen Bioscience | Cat#347526 |
| Rabbit polyclonal anti-human/mouse-IRF8 | Proteintech | Cat#18977-1-AP |
| Rabbit polyclonal anti-human/mouse-Zbtb46 | Ipodix | Cat#IPDX31657 |
| Rabbit anti-human/mouse Vimentin | Abcam | Cat#ab92547 |
| Mouse monoclonal anti-beta-actin | Zen Bioscience | Cat#T200068-8F10 |
| Mouse monoclonal anti-Histone H3 | Zen Bioscience | Cat#250011 |
| Goat anti-Mouse lgG (H+L), HRP | Zen Bioscience | Cat#511103 |
| Goat anti-Mouse IgG (H+L) Secondary antibody, PE | eBioscience | Cat#M30004-1 |
| **Reagents** |  |  |
| RBC Lysis Buffer (10×) | TONBO | Cat#TNB-4300-L100 |
| Foxp3 / Transcription Factor Fix/Perm Diluent (1×) | TONBO | Cat#TNB-1022-L160 |
| Foxp3 / Transcription Factor Fix/Perm Concentrate (4×) | TONBO | Cat#TNB-1020-L050 |
| Lymphoprep^TM^ | STEMCELL^TM^ Technologies | Cat#7851 |
| Collagenase IV | Gibco | Cat#17104019 |
| Dnase I | Solarbio | Cat#D8071 |
| 0.25% trypsin and EDTA  solution | Gibco | Cat#25200072 |
| Percoll | GE Healthcare | Cat#17089109 |
| Streptavidin Particles | BD Biosciences | Cat#557812 |
| Concanavalin A | Sigma-Aldrich | Cat#C5275 |
| Hyaluronidase | Sigma-Aldrich | Cat#H1115000 |
| Phorbol-12-myristate-13-acetate | Alomone Labs | Cat#P-800 |
| Ionomycin | Alomone Labs | Cat#I-700 |
| Brefeldin A | Alomone Labs | Cat#B-275 |
| RIPA Lysis Buffer | Beyotime | Cat#P003B |
| RNAEx ZOL Reagent | ECOTOP | Cat#EK-5301 |
| 2 × RealStar Universal SYBR | Genstar | Cat#A308 |
| DAPI Staining Solution | Beyotime | Cat#C1005 |
| 2-Deoxy-D-glucose | MedChemExpress | Cat#HY-13966 |
| Oligomycin A | MedChemExpress | Cat#HY-16589 |
| Puromycin dihydrochloride | MedChemExpress | Cat#HY-B1743A |
| ***Continued*** | | |
| EasySep^TM^ Human PE Positive Selection Kit II | STEMCELL^TM^ Technologies | Cat#18551 |
| EasySep^TM^ Mouse PE Positive Selection Kit II | STEMCELL^TM^ Technologies | Cat#17666 |
| CellTrace^TM^ CFSE Cell Proliferation Kit | Invitrogen | Cat#C34554 |
| HRP substrate | Millipore | Cat#WBKLS0500 |
| Pierce^TM^ BCA Protein Assay Kit | Thermo Scientific | Cat#23227 |
| Nuclear and Cytoplasmic Protein Extraction Kit | Beyotime | Cat#P0028 |
| StarScript II RT Kit | Genstar | Cat#A214 |
| [Lipomaster 3000 Transfection Reagent](https://www.casmart.com.cn/product-details/page/545/760143373) | Vazyme | Cat#TL301-01 |
| [Dual Luciferase Reporter Assay Kit](https://www.casmart.com.cn/product-details/page/545/881661319) | Vazyme | Cat#DL101-01 |
| 2-Deoxy-D-glucose (2-DG) | Selleck | Cat#S4701 |
| poly-D-lysine | Beyotime | Cat#C0312 |
| Seahorse XF Cell Mito Stress Test Kit | Agilent | Cat#103015-100 |
| Seahorse XFe96/XF Pro FluxPakMini | Agilent | Cat#103793-100 |
| Seahorse XF Glycolysis Stress Test Kit | Agilent | Cat#103020-100 |
| Human OGN Elisa Kit | Dogesce | Cat#DG96179Q |
| Human OPN Elisa Kit | Dogesce | Cat#DG10095H |
| Human PTN Elisa Kit | Dogesce | Cat#DG10402H |
| Human XCL1 Elisa Kit | Dogesce | Cat#DC10426H |
| Human TNF-α Elisa Kit | Dogesce | Cat#DG10053H |
| Human INF-γ Elisa Kit | Dogesce | Cat#DG96661Q |
| Human IL-10 Elisa Kit | Dogesce | Cat#DG10495H |
| Mouse OGN Elisa Kit | Dogesce | Cat#DG96195Q |
| Mouse OPN Elisa Kit | Dogesce | Cat#DG30089M |
| Mouse PTN Elisa Kit | Dogesce | Cat#DG96131Q |
| Mouse XCL1 Elisa Kit | Dogesce | Cat#DG30351M |
| Mouse TNF-α Elisa Kit | Dogesce | Cat#DG30048M |
| Mouse INF-γ Elisa Kit | Dogesce | Cat#DG30056M |
| Mouse IL-10 Elisa Kit | Dogesce | Cat#DG30091M |

**Table S11. List of primers used in reverse transcription-quantitative PCR**

| **Gene name** | **Forward primer (5’→3’)** | **Reverse primer (5’→3’)** |
| --- | --- | --- |
| **Human** | | |
| Hu-*β-ACTIN* | TGACGTGGACATCCGCAAAGACC | CTCAGGAGGAGCAATGATCTTGA |
| Hu-*XCR1* | CTGTTCTGCTACGTGGAGATCC | CAGGGCGTATTCTAGCTGCTG |
| Hu-*CMKLR2* | CATCGTCATTTGGTTCACGGG | TCCGCAATGGCTAGATTGAGG |
| Hu-*CCR1* | AAGTCCCTTGGAACCAGAGAGAAG | CCAACCAGGCCAATGACAAA |
| Hu-*XCL1* | AAGGCTCCTTGAGAGCAGTAA | ATTGGTCGATTGCTGGGTTCC |
| Hu-*XCL2* | AGGGAGTGAAGTCTCACATAGG | GTTGGCTTGGTCTGGATCAT |
| Hu-*S100A9* | ATCATCAACACCTTCCACCA | GCTTGTCTGCATTTGTGTCC |
| Hu-*CYBB* | ACCGGGTTTATGATATTCCACCT | GATTTCGACAGACTGGCAAGA |
| Hu-*COX2* | TCCTAACCCTTTTGTCGCCTG | CTGGCGCTCAGCCATACAG |
| Hu-*ARG1* | TCCAAGGTCTGTGGGAAAAG | TGGTTGTCAGTGGAGTGTTG |
| Hu-*SPP1* | TGAGAGCAATGAGCATTCCGATG | CAGGGAGTTTCCATGAAGCCAC |
| Hu-*OGN* | GAGGATAAATACCTGGATGGA | GTGCGTAAAGATAGGCTGATT |
| Hu-*PTN* | CCTCCCTGTCAGGGCGTAAT | GACGGATGACTCACTGGTCTCTTT |
| Hu-*FOXO1* | CTACGAGTGGATGGTCAAGAGC | CCAGTTCCTTCATTCTGCACACG |
| Hu-*FOXO3* | ACGGCTGACTGATATGGCAG | CGTGATGTTATCCAGCAGGTC |
| Hu-*FOXO4* | GGCTGCCGCGATCATAGAC | GGCTGGTTAGCGATCTCTGG |
| Hu-*FOXO6* | GAAGAACTCCATCCGGCACA | CGGGGTCTTCCCTGTCTTTC |
| Hu-*LMNA* | CTTCGCATCACCGAGTCTGA | AGGTCACCCTCCTTCTTGGT |
| Hu-*PLAUR* | CCAGCAGAACATGGACACGA | CACCAGCTCCAGCTCTTCTC |
| Hu-*CASP9* | TCCTCGCTGCATTTCATGGT | GCATCCATCTGTGCCGTAGA |
| Hu-*VEGF* | CTGGAGCGTGTACGTTGGT | TCCCCAAACTCCTGGTCAGA |
| ***Continued*** | | |
| Hu-*IGF2* | ACGCTTTCTGTTTCTCTCCGT | TCCCATTGGTGTCTGGAAGC |
| Hu-*ITGAD* | CAGAAGCAGCCCCATCAGAG | GTGGATTCTTCCTGCCTGCT |
| **Mouse** | | |
| Ms-*β-Actin* | CGTGCGTGACATCAAAGAGAAG | CGTTGCCAATAGTGATGACCTG |
| Ms-*Xcr1* | GCTGGTGACATCATGTGTGTG | CCTTGTGCGAAACAAAGTCCT |
| Ms-*Xcl1* | TTTGTCACCAAACGAGGACTAAA | CCAGTCAGGGTTATCGCTGTG |
| Ms*-S100a9* | AATGGTGGAAGCACAGTTGG | GCTGATTGTCCTGGTTTGTG |
| Ms*-Cybb* | ATGAGGCTGCTCATCCCTTCCTTGA | CTTTTCTCATCTCGTTCTGCCACCT |
| Ms*-Cox2* | TTCAACACACTCTATCACTGGC | AGAAGCGTTTGCGGTACTCAT |
| Ms*-Arg1* | ATTATCGGAGCGCCTTTCTC | ACAGACCGTGGGTTCTTCAC |
| Ms*-Spp1* | TTCACTCCAATCGTCCCTAC | TTAGACTCACCGCTCTTCAT |
| Ms*-Ogn* | TGCTTTGTGGTCACATGGAT | GAAGCTGCACACAGCACAAT |
| Ms*-Ptn* | TGGAGAATGGCAGTGGAGTGT | GGCGGTATTGAGGTCACATTC |
| Ms*-Foxo1* | GGGTTAGTGAGCAGGTTACAC | TCCAATGGCACAGTCCTTATC |
| Ms*-Lmna* | AGTGAGAAGCGCACATTGGA | ACGCAGTTCCTCGCTGTAAA |
| Ms*-Plaur* | GACTACCGTGCTTCGGGAATG | ATGGTCCTGTTGGTCTTTTCG |
| Ms*-Casp9* | CTCCACCTTCCCAGGTTTTGTC | GCCATCCAAGGTCTCGATGTA |
| Ms*-Glut1* | CTCCACCTTCCCAGGTTTTGTC | GCCATCCAAGGTCTCGATGTA |
| Ms*-Glut3* | ATGGGGACAACGAAGGTGAC | CAGGTGCATTGATGACTCCAG |
| Ms*-Glut4* | CTCATGGGCCTAGCCAATGC | CCCTGATGTTAGCCCTGAGTA |
| Ms*-Snat2* | TAATCTGAGCAATGCGATTGTGG | AGATGGACGGAGTATAGCGAAAA |
| Ms*-Snat4* | AGAAATTCCAAATACCCTGCCC | GAAGCGTGTTGTTGAATGACAG |
| Ms*-Lat1* | CTACGCCTACATGCTGGAGG | GAGGGCCGAATGATGAGCAG |
| ***Continued*** | | |
| Ms*-Fatp1* | CGCTTTCTGCGTATCGTCTG | GATGCACGGGATCGTGTCT |
| Ms*-Fatp2* | GATGCCGTGTCCGTCTTTTAC | GACTTCAGACCTCCACGACTC |
| Ms*-Fatp4* | ACTGTTCTCCAAGCTAGTGCT | GATGAAGACCCGGATGAAACG |
| Ms*-Vegf* | GCAGCGACAAGGCAGACTAT | AAGAGCCCAGAAGTTGGACG |
| Ms*-Igf2* | GCCTCGTCACTTCTCCTACG | CAGTGTCCAGTGCGTGTTTG |
| Ms*-Itgad* | TCCTCCACATCTCACGTCCT | GCTAGCCCAGAGCTGAAGAG |

**Table S12. List of primers used in chromatin immunoprecipitation followed by quantitative PCR (ChIP-qPCR)**

| **Gene name** | **Forward primer (5’→3’)** | **Reverse primer (5’→3’)** |
| --- | --- | --- |
| Hu*-LMNA-*site1 | GACAATCCAACTTTGGGG | GGTCCTGTGGTCACCCAC |
| Hu*-LMNA-*site2 | GGGAAGAAAGCGAGATAGAAA | GGGAGTCGGGTTACAGGGT |
| Hu*-LMNA-*site3 | GCGGGCAAGAAGGGAAGA | CGTGGGCAGGCAAGGTAA |
| Hu*-PLAUR-*site1 | TCCACTGCTGTAAAATGA | AATCCTGTTAGCCAAATA |
| Hu*-PLAUR-*site2 | GGAGGCTGAGGCAGGAGAA | TGCAGTGGCACGAT |
| Hu*-PLAUR-*site3 | TTGAGTAGAAAATAAAGGC | TGATACAACCATTTGGCT |
| Hu*-CASP9-*site1 | ATTCGCCCAGTAGACGCC | ACAAGCCCGAGGGGAGTC |
| Hu*-CASP9-*site2 | AACAAAACAAAATCCCTG | TATCACCTGATCCTTCCA |
| Hu*-CASP9-*site3 | TCATTGGCTCAAAGAAAGT | AGGGATAAGGCACAGGTA |
| Ms*-Lmna--*site1 | GGGTTCTAAGTTATCTGTG | GGTTGGCAAGTCCTATGT |
| Ms*-Lmna-*site2 | CACTCCCTGTCAACTGGT | ACAGGTCAAGAGGTGGAG |
| Ms*-Lmna-*site3 | CTCAAAGGAAATTGCTAC | TTGTTTACTGTGGATGGG |
| Ms*-Plaur-*site1 | GAGGTTCCTGAGTTCAAG | CTATGAAGACCAGCCTTG |
| Ms*-Plaur-*site2 | AGGAGGTTCCTGAGTTCA | CTATGAAGACCAGCCTTG |
| Ms*-Plaur-*site3 | TCTCCCTACAGTTTGTTC | ACAAGTTGAAAGAGGAAC |
| Ms*-Casp9-*site1 | TGTTTTGGATTGATTTTCTG | CAAATTGGTCTGCTGGTG |
| Ms*-Casp9-*site2 | TGCTCCTCCCGAGTGTAC | GATTTGGGCATCAGTCAA |
| Ms*-Casp9-*site3 | GTGGTTTTCTTCCCTTTT | GACCCTACTTCTACAGATACTC |

**Table S13. List of sequences used in dual-luciferase assay**

| **Gene name** | **Forward primer (5’→3’)** | **Reverse primer (5’→3’)** |
| --- | --- | --- |
| *Lmna*-promoter | CCAAGCTTGCATGCCAAGGCCTGGGACTCATTCC | GAGCTCGGTACCCGGTTTCCGGCCATTTTTCTCAGCC |
| *Plaur*-promoter | CCAAGCTTGCATGCCCACCGAGAAAAGCAATACATACCTATTT | GAGCTCGGTACCCGGTTGCTAAAGAGTGAGTCAGCTCCAG |
| *Casp9*-promoter | CCAAGCTTGCATGCCCTCACCGTGTAATCCTCGCT | GAGCTCGGTACCCGGACACCTCCCTCAACGC |
| pCDNA3.1-3×HA-FOXO1-CDS | ATCAGCGGAAAAGCTTGGTACCGAGCTCGGATCCATGGCCGAAGCGCC | TAAACGGGCCCTCTAGACTCGAGTTAGCCTGACACCCAGCTGT |
| pCDNA3.1-Flag-XCR1-CDS | TAGTCCAGTGTGGTGGAATTCATGGAGTCCTCTACAGCCTTTT | TTAAACGGGCCCTCTAGACTCGAGTCAGTAGAAGGAGGGTCCC |
| pCDNA3.1-Flag-XCL1-CDS | AGTCCAGTGTGGTGGAATTCATGAGACTTCTCCTCCTGACTTTCC | GCCCTCTAGACTCGAGTTACCCAGTCAGGGTTATCGCT |
| si-FOXO1 | GGAUUGAACCAGUAUAACU(dT)(dT) | AGUUAUACUGGUUCAAUCC(dT)(dT) |
